# NanoSplicer: accurate identification of splice junctions using Oxford Nanopore sequencing

**DOI:** 10.1093/bioinformatics/btac359

**Published:** 2022-05-27

**Authors:** Yupei You, Michael B Clark, Heejung Shim

**Affiliations:** School of Mathematics and Statistics/Melbourne Integrative Genomics, The University of Melbourne, Melbourne, VIC 3010, Australia; Centre for Stem Cell Systems, Department of Anatomy and Physiology, The University of Melbourne, Melbourne, VIC 3010, Australia; School of Mathematics and Statistics/Melbourne Integrative Genomics, The University of Melbourne, Melbourne, VIC 3010, Australia

## Abstract

**Motivation:**

Long-read sequencing methods have considerable advantages for characterizing RNA isoforms. Oxford Nanopore sequencing records changes in electrical current when nucleic acid traverses through a pore. However, basecalling of this raw signal (known as a squiggle) is error prone, making it challenging to accurately identify splice junctions. Existing strategies include utilizing matched short-read data and/or annotated splice junctions to correct nanopore reads but add expense or limit junctions to known (incomplete) annotations. Therefore, a method that could accurately identify splice junctions solely from nanopore data would have numerous advantages.

**Results:**

We developed ‘NanoSplicer’ to identify splice junctions using raw nanopore signal (squiggles). For each splice junction, the observed squiggle is compared to candidate squiggles representing potential junctions to identify the correct candidate. Measuring squiggle similarity enables us to compute the probability of each candidate junction and find the most likely one. We tested our method using (i) synthetic mRNAs with known splice junctions and (ii) biological mRNAs from a lung-cancer cell-line. The results from both datasets demonstrate NanoSplicer improves splice junction identification, especially when the basecalling error rate near the splice junction is elevated.

**Availability and implementation:**

NanoSplicer is available at https://github.com/shimlab/NanoSplicer and archived at https://doi.org/10.5281/zenodo.6403849. Data is available from ENA: ERS7273757 and ERS7273453.

**Supplementary information:**

Supplementary data are available at *Bioinformatics* online.

## 1 Introduction

Splicing is an essential mechanism in eukaryotic cells that removes introns from pre-mRNAs to create mRNA. Alternative splicing varies which sequences are exonic, enabling a single gene to produce multiple mRNA products (isoforms). Almost 95% of human genes (Pan *et al.*, 2008) undergo alternative splicing, creating a diverse set of transcript isoforms whose expression can control cell functions in a particular condition or developmental stage. Short-read sequencing technologies (e.g. Illumina) successfully identify and quantify local splicing events, such as exon skipping. However, their read lengths (∼150 nt) are much shorter than transcript lengths, making it difficult to combine each splicing event and identify the full-length isoform(s) present (LeGault and Dewey, 2013; Steijger *et al.*, 2013). As such our understanding of the isoform repertoire expressed in different organisms and those that control cell functions remains incomplete.

**Fig. 1.**
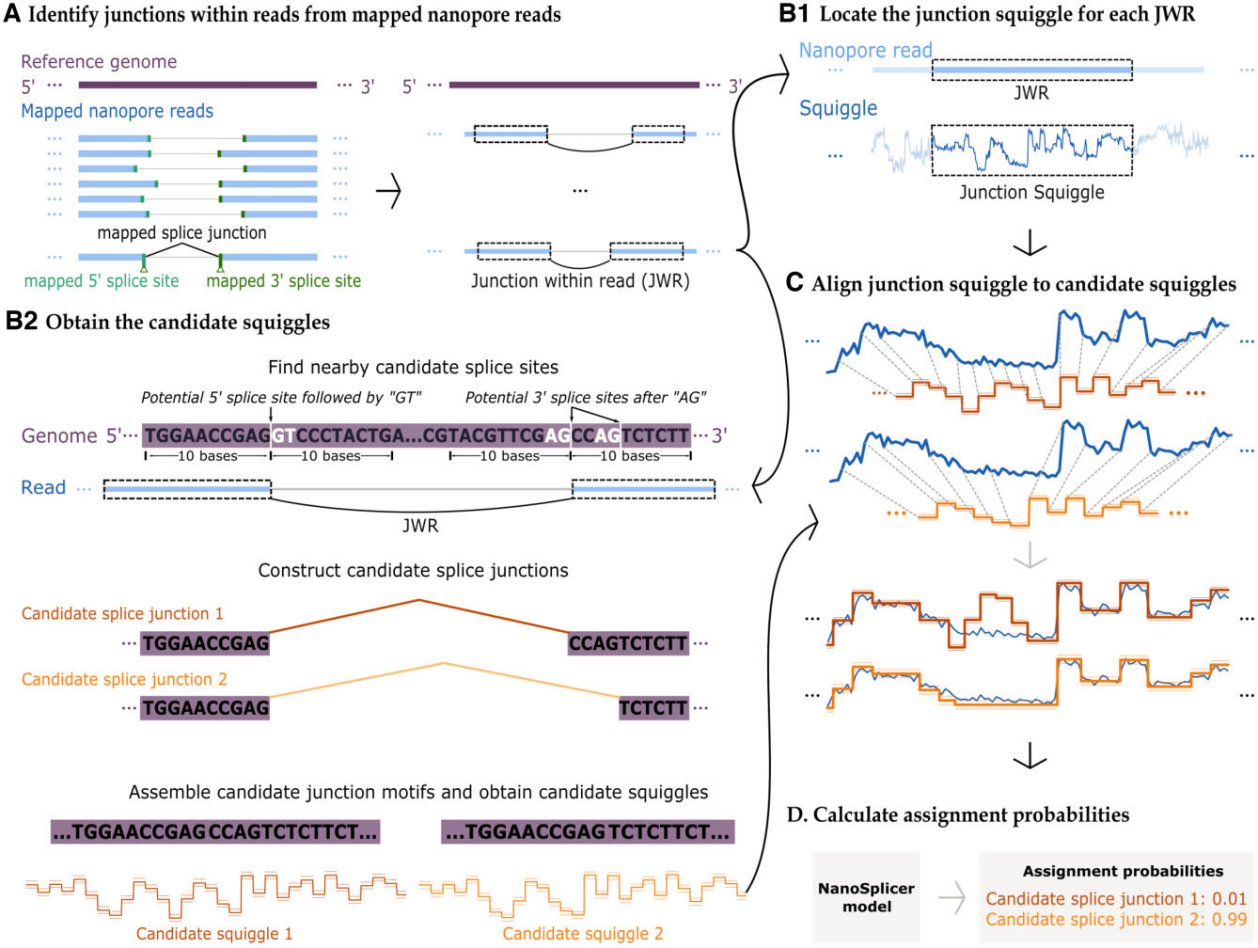
NanoSplicer workflow. (**A**) Identify junctions within reads (JWRs). The left panel shows an example of inconsistently mapped splice junctions in nanopore reads, which may require correction. A splice junction refers to a pair of 5′ and 3′ splice sites, which are the boundaries between introns and exons (shown in green in the figure). Right panel: NanoSplicer locates JWRs in mapped nanopore reads. The two dotted boxes connected by a black curve show a JWR, which is a subsequence of the read that is split and mapped to different exons. (**B1**) Identification of *junction squiggles*. A basecalled nanopore read and its matched raw squiggle are aligned and the portion of the squiggle corresponding to the JWR (dotted boxes) is obtained. (**B2**) Prediction of candidate squiggles. NanoSplicer identifies all possible canonical (‘GT-AG’) splice junctions within 10 bases of the mapped splice sites. Possible 5′ and 3′ splice site nucleotides shown in white. Two candidate splice junctions (1 and 2) are shown (red and orange lines). Candidate junction motifs surrounding the splice junctions are then obtained using the reference genome and candidate squiggles for these motifs predicted with Tombo. Candidate squiggles include predicted mean current (solid line) ±1 standard deviation (dotted line). (**C**) Alignment of candidate and junction squiggles. Top: The junction squiggle (blue) is aligned to each candidate squiggle (red/orange) using dynamic time warping. Dotted lines show which locations of the two squiggles are aligned. Bottom: Each current measurement in the junction squiggle (blue) is shown vertically aligned with its corresponding mean-standard deviation in the candidate squiggle. (**D**) The NanoSplicer model provides assignment probabilities for each candidate by quantifying the squiggle similarity of each alignment (A color version of this figure appears in the online version of this article.)

Nanopore sequencing by Oxford Nanopore Technologies (ONT) is a long-read sequencing method that can connect splicing events by sequencing full-length transcripts (Bolisetty *et al.*, 2015; De Paoli-Iseppi *et al.*, 2021). Nanopore sequencing works by recording changes in electrical current when a DNA or RNA molecule traverses through a pore. This raw signal (known as a *squiggle*) is then basecalled by computational methods, yielding reads that can cover the entire transcript and identify the expressed isoform. However, nanopore reads have a considerably higher basecalling error rate (∼1–10%) and a generally lower throughput than short reads, making their analysis challenging. In particular, the former makes read mapping near splice sites difficult (Tang *et al.*, 2020; Volden *et al.*, 2018; Weirather *et al.*, 2017), making it challenging to distinguish real splice junctions from mapping errors (Fig. 1A). Incorrect detection of splice junctions results in the identification of non-existent isoforms and omission of real isoforms, which inhibits the study of encoded proteins and isoform functions. In this article, we develop a method to accurately identify splice junctions using nanopore sequencing, the performance of which is independent of sequencing throughput.

Several authors have developed methods that correct splice junctions from mapped long reads. Methods such as FLAIR (Tang *et al.*, 2020) and TranscriptClean (Wyman and Mortazavi, 2019) require a set of splice junctions, either from annotations or from matched short reads, to be provided to guide their corrections. However, suitable annotations may not be available, for example, in a non-model organism or in a disease causing altered mRNA processing, while matched short-read sequencing increases costs. Furthermore, in some circumstances, short-reads covering whole transcripts cannot be generated. For example, nanopore sequencing is now being performed on single cells using the popular 10× Genomics platform, however, matched 10× short reads only cover transcript 3′ ends (Lebrigand *et al.*, 2020). Other methods such as StringTie2 (Kovaka *et al.*, 2019), TAMA (Kuo *et al.*, 2020) and 2passtools (Parker *et al.*, 2021) use information from other reads (e.g. nearby splice junctions supported by high read counts) to guide splice junction correction. A limitation with this approach is that it can lead to the replacement of rarer splice junctions with those from more highly expressed isoforms, causing less abundant junctions to go undetected. Moreover, methods that depend on other reads are not well suited to relatively low throughput nanopore datasets, where many isoforms, particularly from lowly expressed genes, have few reads.

The poor performance of nanopore read mapping near splice sites is largely due to basecalling errors, which arise when basecalling methods misinterpret the raw signal squiggles. Motivated by this, here we propose a method, NanoSplicer, which exploits the information in the squiggles to improve splice junction identification. The key idea is to identify, for each splice junction, which of the squiggles predicted from potential splice junction sequences best matches the observed junction squiggle. This ‘squiggle matching’ idea has been successfully applied to map raw signals to a reference genome (Loose *et al.*, 2016; Kovaka *et al.*, 2021; Zhang *et al.*, 2021), and we adapt this idea to develop a method for splice junction identification. By using the squiggle corresponding to each read, NanoSplicer does not require annotations or matched short reads and its performance is not affected by other reads and is independent of read depth, enabling it to identify rare splice junctions. We demonstrate the improved performance of NanoSplicer compared to competing methods using both synthetic and real data. Our method is implemented in the software package NanoSplicer, available at https://github.com/shimlab/NanoSplicer.

## 2 Materials and methods

We developed NanoSplicer to accurately identify splice junctions using nanopore sequencing data. It takes as input mapped nanopore reads, their squiggles and a reference genome sequence. For each read, it outputs lists of candidate splice junctions and the assignment probabilities quantifying the support for each of the candidates. Figure 1 shows an overview of the NanoSplicer workflow. It consists of the following steps:

A. Locate subsequences in the mapped reads which split and map to different exons, supporting potential splice junctions. We refer to these subsequences as *junction within reads (JWRs)*. See Supplementary Section S1.1 for further details regarding JWR identification.

For each JWR, we improve splice junction identification as follows:

B1. Obtain the section of the squiggle corresponding to the JWR location, referred to as a *junction squiggle*.

B2. Construct a list of candidate splice junctions, and predict an expected squiggle for each candidate, referred to as a *candidate squiggle*.

C. Align the junction squiggle to each of the candidate squiggles.

D. Use the NanoSplicer model to quantify the support for each candidate squiggle (assignment probability).

NanoSplicer also allows users to provide additional information to guide the choice of candidate splice junctions in step B2 (see Section 2.2).

We discuss steps B–D in detail in the following sections.

### 2.1 Obtaining a junction squiggle

For each JWR, we obtain its *junction squiggle*, i.e. the squiggle section corresponding to the location of the JWR, as follows. First, we use the ‘resquiggle’ tool in Tombo (Stoiber *et al.*, 2016) to align the nanopore read containing the JWR with its squiggle. Tombo performs the alignment by assigning current measurements in the squiggle to each base of the read. Then, we extract the part of the squiggle aligned to the JWR.

Tombo normalizes squiggles during the alignment to remove systemic differences in shift (median value) and scale between squiggles (https://nanoporetech.github.io/tombo/resquiggle.html#signal-normalization). This normalization enables the resulting junction squiggles to be comparable to candidate squiggles in Sections 2.3 and 2.4. See Supplementary Section S1.3 for additional squiggle preprocessing.

Basecalling errors create challenges in aligning current measurements to bases within reads and therefore in identifying the squiggle region corresponding to the JWR. However, matching over longer regions allows sub-regions with good alignment to be identified and the approximate position of the JWR to be specified. To implement this we take ∼50 nt of the read as the JWR region, which allows us to identify the corresponding squiggle region even if exact base-current alignment for each nucleotide is not obtained. Rare cases where this process still identifies an incorrect region of the squiggle are filtered out (see Section 2.4.4).

### 2.2 Obtaining candidate squiggles

We obtain candidate squiggles by first constructing a list of candidate splice junctions, and then for each candidate, identifying a candidate *junction motif* and predicting its expected *candidate squiggle* (Fig. 1B2). We discuss each step in this section; see Supplementary Section S1.2 for further details.

**Candidate splice junctions**: NanoSplicer provides multiple options to facilitate the selection of candidate splice junctions for each JWR. This allows users to incorporate pre-existing information regarding splice junction usage (if available). By default NanoSplicer will select:

1. The splice junction supported by the JWR (mapped splice junction).
2. Nearby canonical splice junctions. We define these as introns that start with GT and end with AG (Fig. 1B2), a motif present in over ∼99% of mammalian splice junctions (Burset *et al.*, 2000).

Inputs for each JWR can also include:

1. Annotated splice junctions.
2. User-defined list of candidate splice junctions (e.g. from short-read sequencing).
3. Nearby splice junctions supported by other mapped reads (above a user-specified read count threshold).
4. Nearby GC-AG and AT-AC junctions (the most prevalent non-canonical junctions in mammals (Burset *et al.*, 2000)).

Unless stated otherwise, we used the default option to choose candidate splice junctions in this article. This allows NanoSplicer to identify splice junctions solely from the long-read data and does not require prior annotations or information from other reads. For ‘nearby canonical splice junctions’, NanoSplicer identified all GT and AG sequences within 10 nt of the mapped 5′ and 3′ splice sites, respectively and included the splice junctions these would create as candidates.

**Candidate junction motifs:** Once we construct a list of candidate splice junctions, we assemble a *junction motif* for each candidate by connecting sequences from each side of the candidate splice junction using the reference genome. Each candidate junction motif for a JWR extends 5′ and 3′ from the candidate splice junction to a common location. This ensures each candidate has the same nucleotide sequence (and squiggle signal) at the beginning and end, ensuring differences between candidate squiggles are solely due to the various splice junctions utilized.

**Candidate squiggles:** We predict a *candidate squiggle* for each candidate junction motif using an ‘expected current level model’ in Tombo (Stoiber *et al.*, 2016). This model provides the mean and standard deviation of the current level for each nucleotide in a candidate junction motif (https://nanoporetech.github.io/tombo/model\_training.html describes how Tombo computes these). The *candidate squiggle* can then be visualized by fitting a line through the mean for each nucleotide.

### 2.3 Aligning the junction squiggle to each candidate squiggle

For each JWR, we now have its junction squiggle (Section 2.1) and candidate squiggles (Section 2.2). Before measuring the similarity between each candidate and junction squiggle, we first align them, i.e. assign current measurements in the junction squiggle to each mean and standard deviation in the candidate squiggle, so that their time axes are comparable (Fig. 1C). We adapt Dynamic Time Warping (DTW) (Sakoe and Chiba, 1978) to align the two squiggles. DTW is an efficient algorithm for aligning two sequences which may vary in speed; see Keogh and Ratanamahatana (2005) for background on DTW. Supplementary Section S1.4 describes our implementation of DTW which makes the following modifications.

1. We treat the junction squiggle as observations from a model that has the means and standard deviations of each candidate squiggle as parameters. Then, we use the support in the junction squiggle for the model as a measure of similarity in DTW.
2. A single observation has only one mean-standard deviation in a model. Thus, we assign each measurement in the junction squiggle to only one mean-standard deviation in the candidate squiggle.
3. In practice, the start and end of the junction squiggle may not perfectly match that of the candidate squiggles. Thus, we include more nucleotides on each side of the candidate junction motif as a buffer, and then allow the junction squiggle to be aligned to a part of the candidate squiggle.
4. The junction squiggle alignment is expected to cover most nucleotides in the candidate junction motif. Thus, we prevent current measurements in the junction squiggle from being aligned to only a small proportion of the candidate squiggle.

### 2.4 Nanosplicer model: identification of splice junctions

Suppose, for a given JWR, we have its junction squiggle, *M* candidate squiggles, and *M* alignments, each of which aligns the junction squiggle to each candidate squiggle. Let $x=(x_{1},\ldots ,x_{K})$ denote a junction squiggle with length *K*, where *x_k_* is the *k*-th current measurement. The *m*-th candidate squiggle, $c^{m}$, is the sequence of the mean-standard deviation of the current level for each nucleotide in its junction motif. The *M* alignments can be represented by an *M *×* K* matrix $A=\left[a_{mk}\right]$, where $a_{mk}$ indicates the index of the mean-standard deviation in $c^{m}$ where *x_k_* is aligned. Supplementary Figure S5 provides a toy example.

#### 2.4.1 Junction squiggle segmentation

Motivated by basecalling methods (Rang *et al.*, 2018), we partition ***x*** into multiple segments, combine noisy measurements of ***x*** into a more stable summary value (e.g. mean, median) at each segment, and use the summary values as data in our NanoSplicer model (Section 2.4.2). Specifically, we define a segment of ***x*** as consecutive measurements whose alignments to the *M* candidate squiggles (the columns of ***A***) are the same; see Supplementary Figure S5 for a toy example and Supplementary Section S1.6 for our practical implementation of the segmentation. Suppose we have *N* segments in ***x***. Then, we compute the summary $y=(y_{1},\ldots ,y_{N})$, where *y_i_* summarizes information in ***x*** at its *i*-th segment. In this article, we use medians for the summary as they are relatively robust to outliers.

We then compute the candidate squiggles and alignments of ***y*** (denoted by $c_{s}^{1},\ldots ,c_{s}^{M}$ and $A_{s}$, respectively) from that of ***x***; see Supplementary Section S1.6 for details. The NanoSplicer model for ***y*** uses $c_{s}^{1},\ldots ,c_{s}^{M}$ as model parameters and $A_{s}$ provides alignments between ***y*** and the model parameters.

#### 2.4.2 Nanosplicer model

For a given JWR, we build a mixture model to identify a splice junction among the *M* candidates. We introduce a latent variable z∈{1,…,M} indicating which candidate the junction squiggle came from. The mixture model for $y=(y_{1},\ldots ,y_{N})$ can be written as

(1) $$\text{P}(y|\Theta )=\sum_{m=1}^{M}\text{P}(y|z=m,\Theta )\text{P}(z=m|\Theta ),$$

where $\Theta =(c_{s}^{1},\ldots ,c_{s}^{M},A_{s})$. We assume that $y_{1},\ldots ,y_{N}$ are independent conditional on their means and standard deviations, yielding

(2) $$\text{P}(y|z=m,\Theta )=\prod_{n=1}^{N}N^{*}(y_{n};\mu_{i}^{m},\sigma_{i}^{m}),$$

where $\mu_{i}^{m},\;\sigma_{i}^{m}$ are the mean and standard deviation in the candidate squiggle $c_{s}^{m}$ aligned to *y_n_* through $A_{s}$ (i.e. $i=a_{mn}^{s}$). We model *y_n_* using modified normal distributions (denoted by $N^{*}$) which have flat tails, making our method robust to measurements that match none of the *M* candidate squiggles; see Supplementary Section S1.5 for details. Such measurements could appear, for example, due to genetic variants which are not currently incorporated into our junction motifs (see Section 2.2).

When there is other information reflecting the propensity of a candidate to be a splice junction, we can model the mixing proportion P(z=m|Θ) as a function of that information (e.g. nucleotide composition near splice sites for eukaryotes (Irimia and Roy, 2008); see Section 4 and Supplementary Section S1.8). Otherwise, $\text{P}(z=m|\Theta )=\frac{1}{M}$.

#### 2.4.3 Identification of splice junctions

Identification of splice junctions can be performed by computing the posterior probability for each JWR:

(3) $$\begin{array}{c} \text{P}(z=m|y,\Theta )=\frac{\text{P}(y|z=m,\Theta )\text{P}(z=m)}{\sum_{m^{\prime }=1}^{M}\text{P}(y|z=m^{\prime },\Theta )\text{P}(z=m^{\prime })} \end{array}$$

We call this the *assignment probability* that quantifies the support of the junction squiggle for each candidate. In practice, we restrict our identification to JWRs where a single candidate has strong support (e.g. we required an assignment probability > 0.8 in this article).

#### 2.4.4 Squiggle information quality

In practice, we implement the following step to improve performance: the NanoSplicer model assumes that the junction squiggle corresponds to the location of the JWR; however, Tombo can potentially align the JWR to an incorrect squiggle location. Therefore, we add a step to filter out junction squiggles that do not have a high-quality alignment to any candidate squiggle, suggesting they emanate from an off-target read subsequence. First, we measure the alignment quality between a junction squiggle and each of its candidate squiggles using the average log likelihood over the nucleotides of the candidate squiggle; see Supplementary Section S1.7 for details. Then, we compute the maximum of these alignment qualities across *M* candidates, referred to as *squiggle information quality (SIQ)*. In practice, we restrict our splice junction identification to JWRs with SIQ bigger than a threshold to ensure their junction squiggles have high-quality alignments at least one of their candidate squiggles. To choose a suitable threshold, we use an empirical distribution of SIQ constructed by pooling SIQ values from multiple JWRs in the analysis. Assuming that most JWRs are well aligned to correct squiggle locations, we choose an SIQ threshold that identifies junction squiggles whose SIQ values are much smaller than the majority of SIQs in the distribution. We illustrate our choice of thresholds in Sections 3 and 4. See Supplementary Section S2.10.1 for a discussion on how the choice of thresholds involves trade-offs between accuracy and the ability to identify splice junctions. Although we utilize this SIQ step to filter out junction squiggles from off-target regions, it also helps remove poor quality junction squiggles due to experimental artifacts (e.g. current spikes, pore blockages, or uneven dwell time of nucleotides in the pore, etc.) as they can also lead to poor alignments.

## 3 Synthetic RNA data analysis

A potential advantage of NanoSplicer is that it can exploit the information in squiggles. To assess the benefit of this feature, we compared the *accuracy*, defined as the proportion of correctly identified splice junctions, of NanoSplicer to the initial mapping results. We assessed the performance of NanoSplicer using *sequin* RNA standards (Hardwick *et al.*, 2016). Sequins are a set of synthetic spliced mRNA isoforms whose sequences and quantities are precisely known. An *in-silico sequin chromosome* contains each sequin gene and isoform, creating a known ground truth for the position of each splice junction, which mapping- and NanoSplicer-based results can be compared to. Sequins contain 160 isoforms from 76 genes and 745 splice junctions (all but 3 are canonical GT-AG junctions). We used a nanopore sequins cDNA dataset generated using the ONT GridION platform with a R9.4.1 MinION flowcell from Dong *et al.* (2021); see Supplementary Section S2.1 for details of the data. We basecalled raw signals (squiggles) using Guppy 3.6.1 and mapped basecalled reads to the sequin genome using minimap2 (Li, 2018), resulting in 1 919 600 mapped reads to 76 genes and 4 320 441 JWRs. We deactivated the ‘splice flank’ sequence preference option in minimap2 as this preference is not present in sequins. Supplementary Sections S2.2 and S2.3 provides Guppy and minimap2 command lines for our analyses.

For the purpose of assessment, we first assigned one of the 745 sequin splice junctions as the ground truth for each JWR as follows. We mapped the reads to the sequin isoforms using minimap2, providing a 1-1 correspondence between each read and a sequin isoform (Supplementary Section S2.3.2). We restricted our assessment to 1 525 817 reads with the maximum mapping quality (mapQ = 60 in minimap2), for which we can accurately identify their corresponding isoforms. These reads contained 3 526 941 JWRs. Then, on the strand the isoform maps to, we searched for a sequin splice junction whose splice sites are within 10 bases of the mapped JWR splice sites and treated it as a ground truth for that JWR. All the JWRs have either one or zero known sequin splice junctions within 10 bases, supporting their correct assignment. The 190 815 JWRs (5.1%) without a nearby known splice junction have no ground truth and we refer to them as *completely missed JWRs*.

### 3.1 Nanosplicer improves upon the initial mapping results

We used the 3 526 941 JWRs to assess the performance of NanoSplicer (Table 1). The initial mapping failed to identify the ground truths for 286 336 JWRs, (8.1%), including 193 054 completely missed JWRs and 93 282 within 10 bases of the known sequin splice junction. Although any splice junctions identified by NanoSplicer for the completely missed JWRs will be incorrect, we include them in the analysis to assess how well the SIQ (Section 2.4.4) and assignment probability (Section 2.4.3) thresholds in NanoSplicer recognize and filter out these JWRs.

**Table 1.** Accuracy of splice junction identification from initial mapping (minimap2) and NanoSplicer for synthetic (Section 3) and biological (Section 4) data

		Total JWRs	Correct	Incorrect	Accuracy
*Synthetic data*	**Initial mapping**	3 526 941	3 240 605	286 336	91.9%
	**NanoSplicer**	3 066 881	2 934 832	132 049	95.7%
*Biological data*	**Initial mapping**	1 880 011	1 797 512	82 499	95.6%
	**NanoSplicer**	1 724 883	1 674 133	50 750	97.1%

*Note*: NanoSplicer ‘Total JWRs’ are analyzable JWRs (Supplementary Section S2.7) with SIQ >−0.8 and strongest assignment probability >0.8.

We applied NanoSplicer to the dataset to improve upon the initial mapping results; see Supplementary Section S2.14 for NanoSplicer run time. We chose candidates using the default option in Section 2.2; used a uniform prior for mixing proportion (Section 2.4.2); chose −0.8 as a threshold for SIQ using an empirical distribution in Supplementary Figure S10 and required a strongest assignment probability of >0.8. This meant all JWRs had the ground truth as a candidate, except for the completely missed JWRs, as well as JWRs where the ground truth was non-canonical and was not identified as the mapped splice junction. NanoSplicer reported identified splice junctions for 3 066 881 JWRs with an accuracy of 95.7% (see Supplementary Section S2.11 for an explanation of the 4.3% incorrectly identified JWRs). Therefore, NanoSplicer improved the overall accuracy of splice junction detection compared to the initial mapping.

### 3.2 Nanosplicer improvement is greatest when junction alignment quality (JAQ) is low

To better understand the advantages of NanoSplicer we next asked under what circumstances it improved splice junction detection. Basecalling errors can result in low-quality alignments between the JWR and the reference genome. Therefore, we hypothesized that the initial mapping would perform poorly for JWRs with high basecalling errors and that the advantage of NanoSplicer will be greatest for these. We quantified the *junction alignment quality (JAQ)* for each JWR, which we defined as the percentage of matched bases in its alignment (using 25 bases upstream and downstream of mapped splice sites), to test this. For example, a JAQ of 0.96 can be interpreted as 4% of bases in an alignment being inserted/deleted or mismatched; see Supplementary Section S2.6 for further details.

**Fig. 2.**
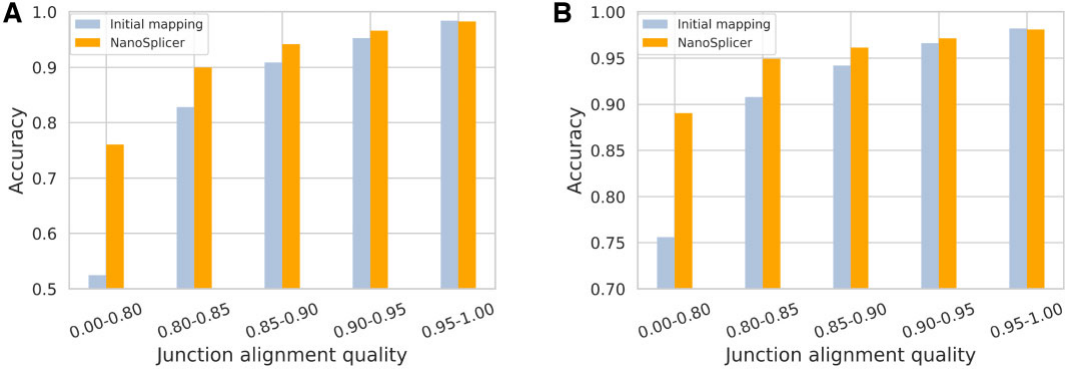
Splice junction identification accuracy of NanoSplicer and minimap2. (**A**) Accuracy of splice junction identification in synthetic data. (**B**) Accuracy of splice junction identification in biological data. A and B: all JWRs were binned based on junction alignment quality (JAQ). The interval ‘a-b’ in the *x*-axis represents ‘a < JAQ ≤ b’. Initial mapping (from minimap2) is based on all JWRs. NanoSplicer accuracy is based on the JWRs where NanoSplicer identifies the splice junction (SIQ > −0.8, strongest assignment probability > 0.8). The number of JWRs in each JAQ bin, including completely missed JWRs, is shown in Supplementary Table S1

Figure 2A shows the accuracy of each approach for JWRs with different ranges of JAQ. NanoSplicer and minimap2 are both similarly accurate (98.3 vs 98.4%) when the JAQ is >0.95 and the JWR sequence aligns almost perfectly. In such a circumstance there is little extra information to be obtained from the squiggle. However, at an alignment quality of 0.95 and below (51.8% of all JWRs), NanoSplicer improves upon the initial mapping accuracy, displaying progressively larger improvements as alignment quality decreases. For junction alignment qualities ≤0.8, NanoSplicer increased the raw accuracy by 23.6% and decreased the proportion of incorrect JWRs by 49.6%. Furthermore, most multi-exon genes have more than one splice junction and JAQ can vary over a read. We find that 20.0% and 77.8% of multi-exon reads have a JWR whose JAQ is ≤0.8 and 0.95, respectively. These results demonstrate that NanoSplicer has the potential to improve splice junction identification in a significant proportion of reads.

While the initial mapping reports splice junctions in all JWRs, NanoSplicer identified splice junctions for 3 066 881 JWRs and its accuracy was evaluated on this smaller set. Thus, multiple factors could contribute to its increased accuracy. These include the ability of SIQ and assignment probability to identify wrongly mapped JWRs, as well as NanoSplicer correction. To investigate the contributions of these factors, we computed the accuracy of the initial mapping on two additional sets of JWRs: the 3 319 482 remaining after SIQ filtering and the 3 066 881 remaining after SIQ and assignment probability filtering prior to NanoSplicer correction (Supplementary Section S2.8 and Table S2). All factors contribute to the increase in accuracy. NanoSplicer correction showed a clear benefit when JAQ ≤ 0.9, while the contribution of SIQ filtering is larger for low-quality alignments (JAQ ≤ 0.8). Moreover, we observed that JWRs filtered by SIQ or assignment probability are enriched in completely missed JWRs (Supplementary Section S2.9), demonstrating these procedures help identify JWRs without true junctions as candidates.

### 3.3 Example of splice junction correction with NanoSplicer

**Fig. 3.**
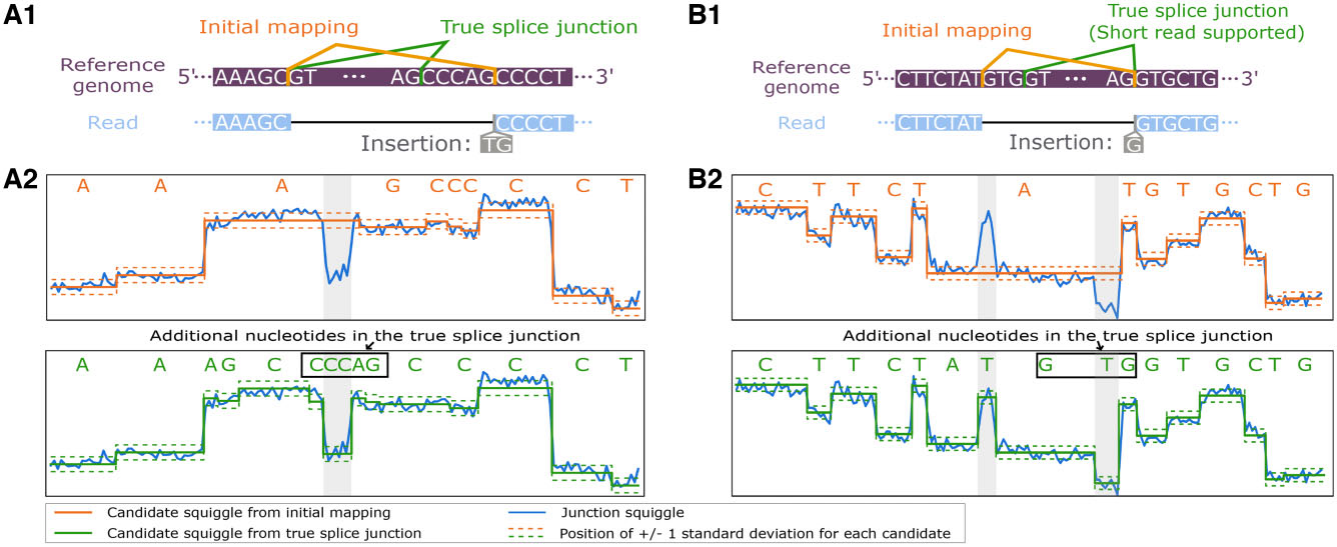
Examples of NanoSplicer correcting wrongly mapped JWRs. (**A1** and **A2**) Synthetic RNA data and (**B1** and **B2**) biological data. (**A1** and **B1**) JWR mapping. Reference genome sequence shown in purple. Green line shows the location of the known ground truth splice junction. The mapped nanopore read (blue) shows the basecalled nucleotides of the JWR and how they were aligned to the reference genome. Orange line shows the splice junction identified by the initial mapping of the JWR. Insertion—basecalled nucleotides in read that were not part of genome alignment. (**A2** and **B2**) Alignment between the junction squiggle for the JWR (blue) and the corresponding candidate squiggles from A1 and B1 (orange and green). Each junction squiggle current measurement is vertically aligned with its assigned mean-standard deviation in the candidate squiggle. The junction motifs for each candidate are shown at the top of each panel. Each nucleotide in the motifs is aligned with its corresponding squiggle position. Panels focus on the junction squiggle areas that distinguish between the candidates (grey background), where the absolute difference in log likelihood between the two candidate models is bigger than 1.35; see Supplementary Section S2.13 for details (A color version of this figure appears in the online version of this article.)

Figure 3A demonstrates how squiggles can provide extra information to identify splice junctions. In the reference genome (Fig. 3A1) there are two ‘AG’ 3′ splice motifs 5 bases apart. The ground truth reveals the upstream site is correct; however, the first 5 exonic bases were basecalled as *‘TG*’ instead of *‘CCCAG*’, causing the JWR to be mapped to the wrong site. We compared the junction squiggle of this JWR to the candidate squiggles obtained from the true splice junction and the initial mapping (Fig. 3A2). The squiggle from the true candidate splice junction is visually a closer match to the junction squiggle. NanoSplicer quantified this squiggle similarity, giving an assignment probability of 0.988 to the true candidate for this JWR. This example demonstrates how nanopore squiggle signal can be used to correct read alignments and accurately identify splice junctions. See Supplementary Section S2.12 for more examples.

## 4 Biological RNA data analysis

In this section, we assess the performance of NanoSplicer on real biological data. We used a nanopore cDNA dataset (see Supplementary Section S2.1 for detailed data description) generated from the lung cancer cell line NCI-H1975, for which short read data is also available (Holik *et al.*, 2017). We basecalled all squiggles using Guppy 3.6.1 and mapped basecalled reads to the human GRCh38 assembly using minimap2. We focused our analysis on 758 009 reads mapped to chromosome 1, yielding 2 220 118 JWRs. Note, we retained the ‘splice flank’ sequence preference option in minimap2 for this analysis.

For the purpose of assessment, we defined a ground truth for each JWR using short-read data from Holik *et al.* (2017), as short reads are expected to accurately identify the locations of splice junctions. We mapped the short reads to GRCh38 using STAR (Dobin *et al.*, 2013) and considered splice junctions with at least three mapped short reads to be ‘known’; see Supplementary Sections S2.4 and S2.5 for details. Then, for each JWR, we searched for a known splice junction within 10 bases of the mapped splice sites and treated it as a ground truth for that JWR. To avoid ambiguity in determining the ground truth, we restricted our assessment to 1 880 011 JWRs that had at most one nearby known splice junction.

### 4.1 Nanosplicer improves upon the initial mapping results

We assessed the performance of NanoSplicer on the 1 880 011 JWRs (Table 1); see Supplementary Section S2.14 for the run time. As previously, we compared the accuracy (i.e. the proportion of correctly identified splice junctions) of NanoSplicer to the initial mapping results. The initial mapping failed to identify the ground truths for 82 499 JWRs (4.4%), including 52 169 completely missed JWRs. In this analysis, we incorporated ‘splice flank’ sequence preferences, like those in minimap2, as prior mixing proportions in NanoSplicer; see Supplementary Section S1.8 for details. Other steps in the NanoSplicer workflow were as per the synthetic data analysis. NanoSplicer identified splice junctions for 1 724 883 JWRs with an accuracy of 97.1%, confirming it improves splice junction detection from biological data (Table 1).

Similar to the synthetic data analysis, the improvement in splice junction identification with NanoSplicer increased as JAQ decreased (Fig. 2B). NanoSplicer improved upon the initial mapping when JAQ ≤ 0.95 and was particularly pronounced below a JAQ of 0.8 (13.4% increase in accuracy, ∼55% decrease in incorrect JWRs).

As the accuracy of NanoSplicer is evaluated on a smaller set of JWRs (1 724 883) than the initial mapping, we again investigated the contributions of SIQ, assignment probability and NanoSplicer correction to the increased accuracy. Results were consistent with the synthetic data analysis: all factors contribute to the increased accuracy; NanoSplicer correction is beneficial when JAQ ≤ 0.95 and SIQ filtering has a relatively larger contribution when JAQ ≤ 0.8 (Supplementary Section S2.8).

### 4.2 Example of splice junction correction with NanoSplicer

Figure 3B shows an example JWR from the biological dataset. Two ‘GT’ splice motifs only 3-bases apart at the 5′ splice site create two candidate splice junctions (Fig. 3B1). One of the candidate splice junctions is supported by the short read data and is assumed to be true. However, the nanopore read in Figure 3B1 was mapped to the other candidate splice junction due to basecalling errors at the 5′ splice site (the *‘GTG*’ bases preceding the splice junction were basecalled as only *‘G*’). We compared the junction squiggle of this JWR to the squiggles obtained from both candidate splice junctions (Fig. 3B2). The shape of the squiggle for the true candidate is clearly a better match for the junction squiggle, while the initial mapping candidate misses clear signal changes indicative of additional bases. NanoSplicer quantified this squiggle similarity, leading to an assignment probability of 0.997 to the true candidate for this JWR. See Supplementary Section S2.12 for more examples.

### 4.3 Identification of non-canonical junctions

A small proportion of splice junctions do not use the canonical GT-AG motif and are challenging to identify from error prone reads. Comparing JWRs from the NCI-H1975 long-reads to the short-read data revealed 8861 (0.47%) had a non-canonical truth. Minimap2 correctly identified 4127 of these (46.6%), while NanoSplicer identified 3661 of 7373 (49.7%) after SIQ and assignment probability filtering. Using the default option NanoSplicer can only consider non-canonical junctions as candidates if they are the mapped splice junctions, meaning it cannot correct the 4734 JWRs where minimap2 was incorrect. However, NanoSplicer provides the option to also include the most common non-canonical splice junctions (GC-AG and AT-AC) as candidates and/or use user supplied lists of candidate splice junctions (e.g. non-canonical splice junctions from GENCODE v39 (Frankish *et al.*, 2021)). Utilizing these options increased accuracy to 83.8% (5300 of 6323, see Supplementary Section S2.15) and 95.9% (7744 of 8071) respectively for JWRs with a non-canonical truth that passed filtering. These results demonstrate the flexible inputs NanoSplicer can utilize and how NanoSplicer performance for non-canonical splice junction identification can benefit from their use.

### 4.4 Improved accuracy when there are rare, unannotated or multiple nearby splice junctions

Methods such as StringTie2 (Kovaka *et al.*, 2019) correct splice junctions using information from other mapped reads, potentially causing less abundant junctions to go undetected. To demonstrate the advantage of NanoSplicer in rare junction identification, we compared NanoSplicer with StringTie2 at all sites where we could ascertain if rarer splice junctions were being replaced. Specifically, we focused on 492 sites where there are multiple nearby splice junctions in use (evidenced from matched short-reads). Among the 492 sites, StringTie2 detected the rarer splice junctions for 10 (2%), while NanoSplicer was successful for 273 (55.5%). This result illustrates the benefits of NanoSplicer, whose performance is independent of other reads, in identifying rare junctions. See Supplementary Section S2.16 for more details and an example.

While NanoSplicer does not require annotations, it provides the option to utilize them in organisms where comprehensive annotations are available. We compared the output of FLAIR (which requires input splice junctions) with NanoSplicer, including human GENCODE v39 annotations as input, to understand the relative performance of each on different categories of JWRs. We find that NanoSplicer provides increased accuracy when there are multiple annotated junctions within 10 nt of the JWR, or the true junction utilized unannotated splice site(s) (Supplementary Fig. S17). NanoSplicer can also be run in ‘annotation only’ mode, which only uses annotated junctions as candidates and hence JWRs can only be corrected to annotated junctions (if present). This mode provides a significant speed increase (∼20 times faster) and outperforms FLAIR when there are multiple nearby annotated junctions while giving equivalent results otherwise (Supplementary Fig. S17). See Supplementary Section S2.17 for further details.

## 5 Conclusion

We have developed a novel method, NanoSplicer, to accurately identify splice junctions using nanopore sequencing. The method, adapting the ‘squiggle matching’ idea, exploits the information in squiggles to improve identification. This enables NanoSplicer to identify splice junctions solely from the nanopore data without requiring annotations or matched short reads. It also enables its performance to be independent of other reads or read depth, having the potential to better identify rare splice junctions. Using both synthetic and real data, we show that NanoSplicer improves upon the initial mapping, particularly when the basecalling error rate near splice junctions is high, demonstrating the contribution of squiggle information to splice junction identification. We also show that NanoSplicer outperforms competing methods when there are rare, unannotated or multiple nearby splice junctions.

To our knowledge, this is the first method that exploits squiggle information for splice junction identification. Therefore, there are many opportunities for potential improvements. First, the NanoSplicer model treats the summary values of junction squiggles as observed data and ignores their uncertainty, possibly leading to a decreased accuracy. One potential way to incorporate the uncertainty is to exploit the likelihood approximation as described in Shim *et al.* (2021), yielding likelihoods expressed by the estimates of model parameters and their standard errors. Second, very short or long dwell times (i.e. the duration of a translocation event) may not reflect typical translocation events (Díaz Carral *et al.*, 2021), potentially causing misleading results. Here, we partly address this issue by filtering out summary values based on a very small or large number of measurements, but a more principled approach, such as modeling dwell time, could potentially improve performance. Third, here we predict expected squiggles from junction motifs using the ‘expected current level model’ in Tombo (Stoiber *et al.*, 2016), but this can be achieved by using other models that are appropriate for the chemistry in use. Indeed, the optimal choice of models may be one learned from the data at hand. Finally, NanoSplicer has been comprehensively tested only for analysis of Nanopore cDNA data and the R9 pore but will be tested on direct RNA sequencing and the R10 pore in the future.

NanoSplicer identifies splice junctions only among candidates, potentially leading to false detection when the true junctions are not included. We are not alone in having this limitation; for example, other tools restrict their correction to junctions from annotations or matched short reads (Tang *et al.*, 2020; Wyman and Mortazavi, 2019), and/or to junctions supported by mapped reads (Kovaka *et al.*, 2019; Kuo *et al.*, 2020; Parker *et al.*, 2021). However, NanoSplicer provides flexible options for candidate selection (Sections 2.2 and 4.3), enabling users to use context-dependent candidates. Moreover, our empirical analysis in Supplementary Section S2.9 shows that SIQ and assignment probability help filter out JWRs without true junctions as candidates, reducing false identifications.

NanoSplicer has been designed and tested for accurate identification of splice junctions. The identified junctions could be leveraged in different types of downstream analyses, including isoform identification and quantification. For example, lists of high confidence splice junctions from NanoSplicer can be inputs into tools such as FLAIR and Stringtie2 to improve their performance. Alternatively, NanoSplicer can examine the read-level support for novel isoforms identified by other tools. For some analyses, however, NanoSplicer’s outputs should be used with care as it identifies splice junctions only for JWRs whose squiggles are informative. For example, if the outputs are used to quantify usage of splice junction(s), excluding JWRs without identified outputs or correcting them using splice junctions from other reads may lead to less accurate quantification because they are not a random subset of all JWRs (Supplementary Fig. S19). Additionally, utilizing mapped junctions from these JWRs to supplement NanoSplicer outputs would decrease overall accuracy as such JWRs tend to have lower JAQs (Supplementary Fig. S20). We are currently investigating to what extent this limitation can be overcome to allow NanoSplicer to accurately quantify splice junctions and produce corrected isoforms instead of corrected JWRs. This would enable NanoSplicer to take full advantage of its potential to improve identification of unannotated, rare or closely related isoforms.

NanoSplicer provides flexible options to be run on JWRs from genes, genomic regions or reads of interest; as well as transcriptome-wide, shortening run time. Our analysis shows that NanoSplicer improvement is greatest when JAQ is low, while initial mapping results tend to be correct for JWRs with high JAQs because their sequences align almost perfectly. In practice, the first step in our software is calculation of JAQs for JWRs, which provides useful and quickly accessible information on long-read junction quality for an experiment or read of interest. Thus, our software offers an option to output JAQs without running the identification step. Additionally, NanoSplicer provides an option to run it on JWRs below a user-specified JAQ threshold (default 0.95) to reduce its run time and focus on the JWRs it is most likely to correct. Nanopore sequencing accuracy is increasing over time, however even as median read accuracy has increased, Nanopore read accuracy distributions still exhibit a long tail of reads with lower accuracy. Therefore, there remains a significant proportion of reads for which splice junction identification (and subsequent isoform identification) can be enabled by NanoSplicer.

## Supplementary Material

btac359_Supplementary_Data
