# Supplementary material for: NanoSplicer: accurate identification of splice junctions using Oxford Nanopore sequencing
Source: Bioinformatics. 2022 May 27;38(15):3741–8. doi: 10.1093/bioinformatics/btac359 (PMC9344838; doi:10.1093/bioinformatics/btac359)
Supplement: btac359_Supplementary_Data [file btac359_supplementary_data.pdf]

Supplementary Material
NanoSplicer: Accurate identification of splice junctions using Oxford Nanopore sequencing

Yupei You [1], Michael B. Clark [2,*] and Heejung Shim [1,*]

[1]School of Mathematics and Statistics and Melbourne Integrative Genomics, The University of Melbourne, Parkville, Australia

[2]Centre for Stem Cell Systems, Department of Anatomy and Physiology, The University of Melbourne, Parkville, Australia.

1 Supplementary Methods

1.1 Junction within reads (JWRs)

Junction within reads (JWRs) are defined as the subsequences in mapped long reads which split and map to different exons, supporting potential splice junctions. Each JWR requires 1. the location of mapped splice junction (a pair of mapped 5' and 3' splice sites) and 2. subsequences from both exons. NanoSplicer takes as input mapped long reads in BAM/SAM format, from which the location of mapped splice junctions can be quickly extracted. Then, we identify subsequences from both exons as follows. To ensure a JWR contains information about all its candidate splice junctions, we choose the subsequences to be included in the JWR after obtaining all candidate junctions. Section 2.2 in the main text describes how NanoSplicer selects candidate splice junctions and supplementary section 1.2.1 provides a detailed procedure to identify nearby canonical candidate splice junctions (one option for candidate splice junctions).

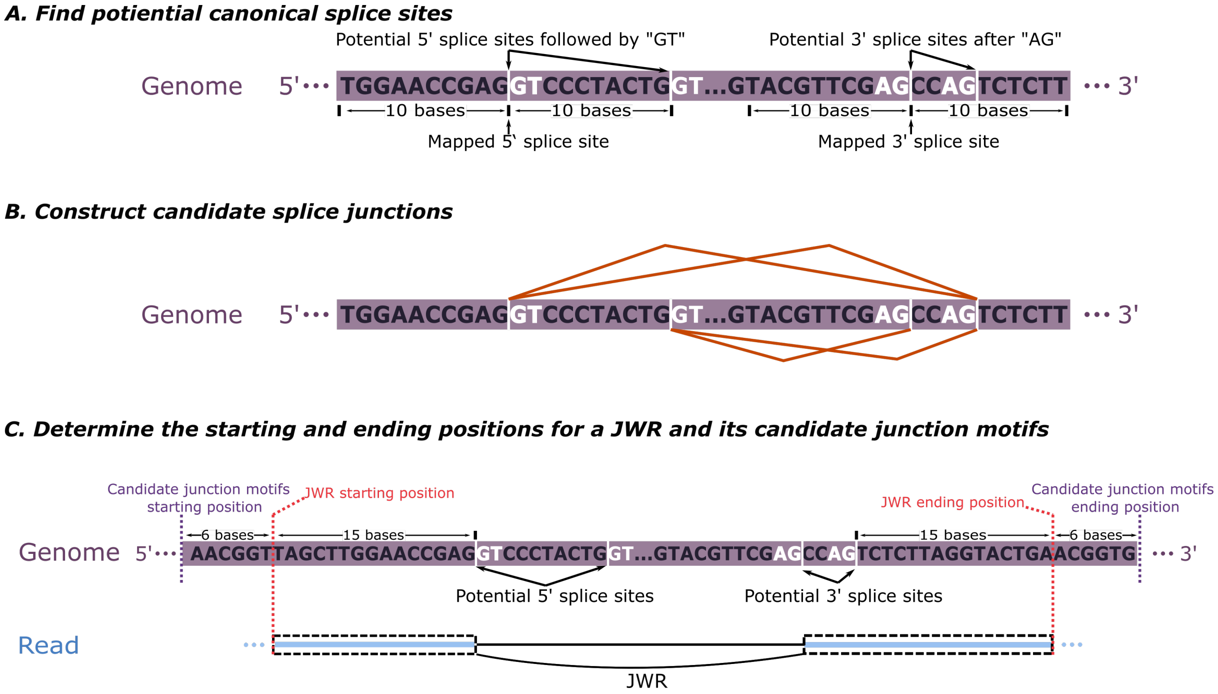

Fig. S1: **Candidate splice junctions, candidate junction motifs, and junction within reads (JWRs)** A: All potential 5’ and 3’ canonical splice sites within 10nt of the mapped splice sites. B: Construct canonical candidate splice junctions by considering all 5’-3’ combinations of the splice sites identified in A. C: Panel shows the start and end positions of a JWR and its candidate junction motifs for a list of candidate splice junctions

Once we obtain all candidate splice junctions, we identify the 5’ and 3’ splice sites farthest away from each other among all 5’ and 3’ splice sites in the candidate junctions (e.g., leftmost 5’ splice site and rightmost 3’ splice site in supplementary Fig. S1C). Then, we use the 15th nucleotide preceding the 5’ splice site and the 15th nucleotide after the 3’ splice site as the first and last nucleotides of the subsequences for the JWR, respectively.

1.2 Obtaining candidate squiggles

Section 2.2 in the main text describes each step of obtaining candidate squiggles: constructing a list of candidate splice junctions, and then for each candidate, identifying a candidate junction motif and predicting its expected candidate squiggle. Here, we will provides details on the iden-

tification of nearby canonical splice junctions (one option for candidate splice junctions) (supplementary section 1.2.1), the selection of the start and end positions of each candidate junction motif (supplementary section 1.2.2), and the prediction of an expected candidate squiggle using the "expected current level model" in Tombo (supplementary section 1.2.3).

1.2.1 Canonical ("GT-AG") splice junctions as candidate splice junctions

For each JWR, to identify nearby canonical splice junctions (introns that start with "GT" and end with "AG"), we first search for all potential 5' splice sites followed by "GT" within 10nt of the mapped 5' splice site and all potential 3' splice sites after "AG" within 10 nt from the mapped 3' splice site (supplementary Fig. S1A). Then, we consider all possible combinations of these potential 5' and 3' splice sites, creating canonical splice junctions (supplementary Fig. S1B).

If the transcript strand information is recorded in the input BAM/SAM file (i.e. 'ts' tag is present in the BAM/SAM file), we search for canonical candidate splice junctions on the transcript strand of the reference genome. Otherwise, the "GT-AG" pattern from both strands will be included as candidates.

1.2.2 Start and end positions of candidate junction motifs

Each candidate junction motif for a JWR extends 5' and 3' from the candidate splice junction to a common location which is the start and end positions of the JWR (see supplementary Fig. S1C and supplementary section 1.1). This ensures each candidate has the same nucleotide sequence (and squiggle signal) at the beginning and end, ensuring differences between candidate squiggles are solely due to the various splice junctions utilised.

In practice, the start and end of the junction squiggle may not perfectly match that of JWR (or start and end positions of candidate junction motifs). Thus, we include 6 nucleotides at each side of the candidate junction motif as a buffer (see supplementary Fig. S1C), and allow the

junction squiggle to be aligned to a part of the candidate junction motifs (see supplementary section 1.4.2).

1.2.3 Prediction of an expected candidate squiggle from a candidate junction motif

Nanopore sequencing works by recording changes in electrical current when a DNA or RNA molecule traverses through a pore. During the transmission of the DNA/RNA molecule, multiple nucleotides occupy the pore simultaneously. Thus, the current level is influenced by a k-mer (consecutive nucleotides with length of k). We predict a candidate squiggle for each candidate junction motif using an "expected current level model" in Tombo v1.5. The expected current level model provides the mean and standard deviation of the current level for each 6-mer; see `https://nanoporetech.github.io/tombo/model\_training.html` for the procedure in Tombo to compute these means and standard deviations.

For simplicity, in the main text, we use the third nucleotide in a 6-mer to represent the whole 6-mer by following Tombo's procedure to match nucleotides to 6-mers. For example, to predict an expected squiggle for a motif *GCAG*, we first include 2 and 3 nucleotides present before and after the motif, respectively, yielding an extended motif, such as, *AGGCAGCTG*. Then, we convert this extended motif into four 6-mers (*AGGCAG, GGCAGC, GCAGCT* and *CAGCTG*), resulting in four mean-standard deviations, each of which is the mean-standard deviation of current levels for each 6-mer.

Note that the means and standard deviations provided by the expected current level model in Tombo are computed using normalised squiggles; see `https://nanoporetech.github.io/tombo/resquiggle.html` for details on the normalisation procedure in Tombo. NanoSplicer obtains junction squiggles using the "resquiggle" tool in Tombo which performs the same normalisation procedure. This enables the junction squiggles to be comparable to candidate squiggles.

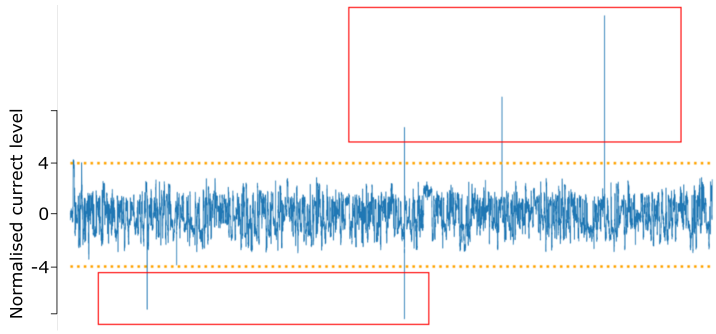

Fig. S2: **Visualisation of a squiggle corresponding to a long read from the synthetic data** The red boxes highlight transient current spikes within the squiggle. The orange lines indicate the threshold ($T = 4$) to remove spikes in our analysis of the paper. Note that the squiggle in this figure is from an entire read.

1.3 Junction squiggle preprocessing

We occasionally observe transient current spikes in junction squiggles which are a few current measurements far away from a typical range of squiggle current measurements (between -3 and 3 for squiggles normalised using Tombo; see `https://nanoporetech.github.io/tombo/resquiggle.html` for details on the normalisation procedure in Tombo). Supplementary Fig. S2 visualises a squiggle corresponding to a long read from the synthetic data (see section 3 in the main text or supplementary section 2.1 for details of the data), showing a typical range of normalised current measurements, as well as examples of the spikes. NanoSplicer removes those spikes from junction squiggles by eliminating current measurements whose absolute values are bigger than a user-specified threshold T. In the analysis of this paper, we used $T = 4$ to remove spikes from junction squiggles which are normalised by Tombo.

1.4 Dynamic time warping (DTW) in NanoSplicer

In NanoSplicer, we adapt DTW to align a junction squiggle to each of its candidate squiggles. DTW is an efficient algorithm for aligning two sequences which may vary in speed. Formal backgrounds on DTW can be found in Keogh and Ratanamahatana (2005). Here, supplementary

section 1.4.1 presents our implementation of DTW in NanoSplicer which places restrictions on standard DTW, and supplementary section 1.4.2 details these restrictions.

1.4.1 Align junction squiggle to candidate squiggles using DTW

NanoSplicer aligns a junction squiggle to each of its candidate squiggles using DTW. For each alignment, NanoSplicer treats the junction squiggle as observations from a model that has the means and standard deviations from each candidate squiggle as parameters. Then, we use a negative log-likelihood as a measure of distance in DTW. Let $\boldsymbol{x} = (x_1, \ldots, x_K)$ be the junction squiggle with length K, where x_k is the k-th current measurement. Let $\boldsymbol{c}^m = (\mu_i^m, \sigma_i^m)_{i=1}^{L_m}$ represent the m-th candidate squiggle, where μ_i^m and σ_i^m are the mean and standard deviation of the current level for the i-th k-mer in its junction motif with length L^m. Note that for simplicity, the main text describes "the mean and standard deviation for each nucleotide" instead of "for k-mer"; see supplementary section 1.2.3 for the definition of k-mer and the relationship between a nucleotide and a k-mer. Our implementation of DTW for the alignment is the same for all M candidate squiggles, so we will describe it for a single candidate squiggle and drop the superscript ^{m} for the rest of supplementary section 1.4.

Let $\boldsymbol{D} = [d_{i,k}]$ represent an $L \times K$ distance matrix, where $d_{i,k}$ is a distance between x_k and (μ_i, σ_i) defined by

$$d_{i,k} = -\log f(x_k|\mu_i, \sigma_i), \tag{1}$$

where f is the probability density function of the modified normal distributions (see supplementary section 1.5 for the definition). Let $\boldsymbol{a} = (a_1, \ldots, a_K)$ denote an alignment between $\boldsymbol{x}$ and $\boldsymbol{c}$, where a_k indicates the index of the mean-standard deviation in $\boldsymbol{c}$ where x_k is aligned. For example, $a_k = j$ indicates that x_k and (μ_j, σ_j) are aligned. Using DTW, NanoSplicer finds the alignment that minimises a distance between $\boldsymbol{x}$ and $\boldsymbol{c}$, defined by $\sum_{k=1}^{K} d_{a_k,k}$, among alignments satisfying the restrictions described in the following section 1.4.2.

1.4.2 DTW restrictions in NanoSplicer

Here, we detail the restrictions that are imposed on alignments when NanoSplicer searches for the alignment with minimum distance between $\boldsymbol{x}$ and $\boldsymbol{c}$. Supplementary Fig. S3 shows examples of a valid alignment (satisfying all restrictions) and an invalid alignment (breaching the restrictions).

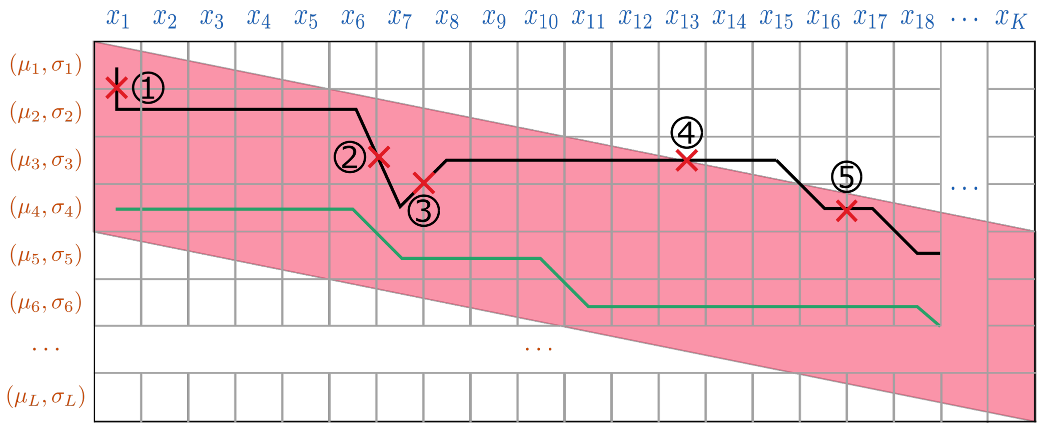

Fig. S3: **Examples of valid and invalid alignments in NanoSplicer** Figure shows an $L \times K$ matrix having junction squiggle $x_1, \ldots, x_K$ in its column and candidate squiggle $(\mu_1, \sigma_1), \ldots, (\mu_L, \sigma_L)$ in its row, respectively. An alignment is represented by a path on the matrix. Specifically, a path passing the grid at i-th row and j-th column indicates that (μ_i, σ_i) and x_j are aligned. The diagonal pink band is the area where valid paths are restricted to be inside. The green path shows a valid path in NanoSplicer DTW implementation. The black line represents an invalid path, and the red "X"s show where each of the restrictions described in supplementary section 1.4.2 is breached: 1) x_1 is aligned to multiple elements in the candidate squiggle; 2) The continuity is breached; 3) The monotonicity is breached; 4) A path is not allowed to go out of the pink band; 5) The minimum dwell time t ($t = 4$ in this example) is not satisfied as the number of current measurements aligned to (μ_4, σ_4) is less than t $(= 4)$.

- **No warping in the junction squiggle:** We treat each current measurement of the junction squiggle as an observation from a model that has means and standard deviations of each candidate squiggle as parameters. Each single observation has only one mean-standard deviation in the model. Thus, each measurement in $\boldsymbol{x}$ is aligned to only one mean-standard deviation in the candidate squiggle.

- **Monotonicity and continuity in the candidate squiggle:** An alignment $\boldsymbol{a} = (a_1, \ldots, a_K)$ is valid only if $a_1, \ldots, a_K$ are monotonically and continuously ordered. That is, $a_k \leq a_{k+1}$ and $a_{k+1} - a_k \leq 1$, for all $k \in \{1, \ldots, K-1\}$.

- **Warping band:** An alignment $\boldsymbol{a} = (a_1, \ldots, a_K)$ can be represented by a path $(a_1, 1), \ldots, (a_K, K)$ in an $L \times K$ matrix, where L is the length of the candidate squiggle. Paths are constrained to be within a diagonal band with bandwidth $w \times L$, where $w \in (0, 1]$ is a user-specified value. See supplementary Fig. S3 for examples of paths and band. Specifically, (a_k, k) has to be within the band for all $k \in \{1, \ldots, K\}$. In our paper, we used $w = 0.4$. The motivations of this warping band restriction include:

  - The junction squiggle alignment is expected to cover most nucleotides in the candidate junction motif. Thus, we prevent current measurements in the junction squiggle from being aligned to only a small proportion of the candidate squiggle.
  - In practice, the start and end of the junction squiggle may not perfectly match that of the candidate squiggles. Thus, we include more nucleotides at each side of the candidate junction motif as a buffer (see supplementary section 1.2.2 for more details), and then allow the method to search for the best start and end positions of the junction squiggle alignment among that near the two ends of the candidate squiggle (i.e., path does not have to start at $(1, 1)$ or end at (L, K) on the matrix.).
  - It speeds up the DTW algorithm which searches for the best alignment (see Keogh and Ratanamahatana (2005) for more explanation).

- **Minimum dwell time:** Very short dwell times (i.e., the duration of a translocation event) may not reflect typical translocation events, potentially causing misleading results in NanoSplicer. Thus, NanoSplicer restricts the number of current measurements in $x_1, \ldots, x_K$

aligned to (μ_j, σ_j) to ones bigger than or equal to t for all $j \in 1, \ldots, L$. We used $t = 4$ in the analysis of the paper.

1.5 Modified normal distributions

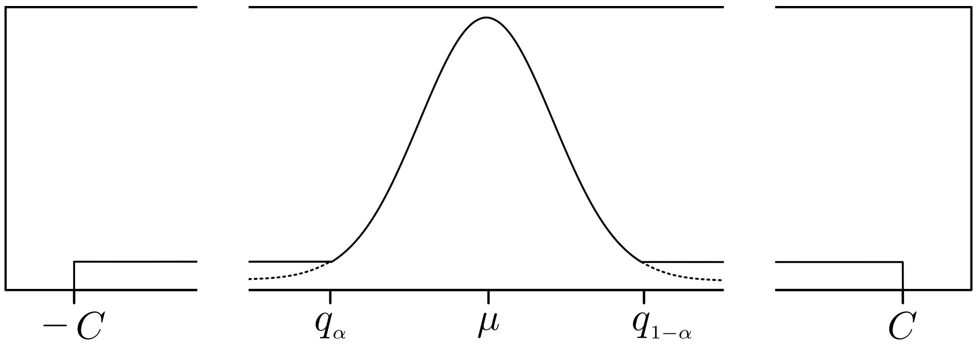

Fig. S4: **Modified normal distribution** The probability density function of the modified normal distribution has flat tails.

In our NanoSplicer model in section 2.4.2 in the main text, we used modified normal distributions which have flat tails, making our methods robust to measurements that match none of the M candidate squiggles. This section describes the modified normal distribution with μ, σ, C, and α as parameters. The probability density function of the modified normal distribution can be represented by:

$$f(y|\mu, \sigma, C, \alpha) \propto \begin{cases} \frac{1}{\sqrt{2\pi\sigma^2}} e^{-\frac{1}{2}\left(\frac{y-\mu}{\sigma}\right)^2}, & \text{if } y \text{ in } [q_\alpha, q_{1-\alpha}], \\ \frac{1}{\sqrt{2\pi\sigma^2}} e^{-\frac{1}{2}\left(\frac{q_\alpha-\mu}{\sigma}\right)^2}, & \text{if } y \text{ in [-C,} q_\alpha \text{) or (} q_{1-\alpha} \text{,C],} \\ 0, & \text{otherwise,} \end{cases} \tag{2}$$

where q_α and $q_{1-\alpha}$ are the α and $1-\alpha$ quantiles of the normal distribution with mean μ and standard deviation σ. Supplementary Fig. S4 shows the shape of this density function. In the analysis of this paper, we used $\alpha = 0.01$ (see supplementary section 2.10.2 for the performance assessment with different values of α) and $C = 4$ to ensure all observed current measurements and their summary values are between $-C$ and C.

1.6 Junction squiggle segmentation

Section 2.4.1 in the main text describes NanoSplicer procedure for junction squiggle segmentation, supplementary Fig. S5 provides a toy example, and supplementary Fig. S6A visualises the segments of a junction squiggle for 3 candidates. Here, we will describe our procedure to compute the candidate squiggles and alignments of the summary $\boldsymbol{y}$ (denoted by $\boldsymbol{c}_s^1, \ldots, \boldsymbol{c}_s^M$ and $\boldsymbol{A}_s$, respectively) from that of $\boldsymbol{x}$ (supplementary sections 1.6.1 and 1.6.2) and our practical implementation of the segmentation (supplementary section 1.6.3).

1.6.1 Candidate squiggles for the summary from junction squiggle segments

The standard deviations of the summary values are expected to be smaller than that of the original current measurements. Thus, we estimate standard deviations of the summary values from squiggle data, and use them to predict candidate squiggles, $\boldsymbol{c}_s^1, \ldots, \boldsymbol{c}_s^M$, for the summary which have been used as model parameters in the NanoSplicer model. A key idea to estimate standard deviations of the summary is to assume that summary values from the same squiggle (i.e., the same read) share the same standard deviation and to estimate the shared standard deviation by using multiple summary values from an entire squiggle. Specifically, for each (entire) squiggle, we already have its alignment to its basecalled read (from the "resquiggle" tool in Tombo during the step of obtaining junction squiggles; see section 2.1 in the main text). Let L denote the number of nucleotides in the basecalled read. We first partition current measurements in the (entire) squiggle into L sections by using their alignments to the read (i.e., consecutive measurements aligned to the same nucleotide belong to the same section). Then, we compute compute the summary $\tilde{\boldsymbol{y}} = (\tilde{y}_1, \ldots, \tilde{y}_L)$, where $\tilde{y}_i$ summarises information in the squiggle at its i-th section. The standard deviation of the summary values from this squiggle

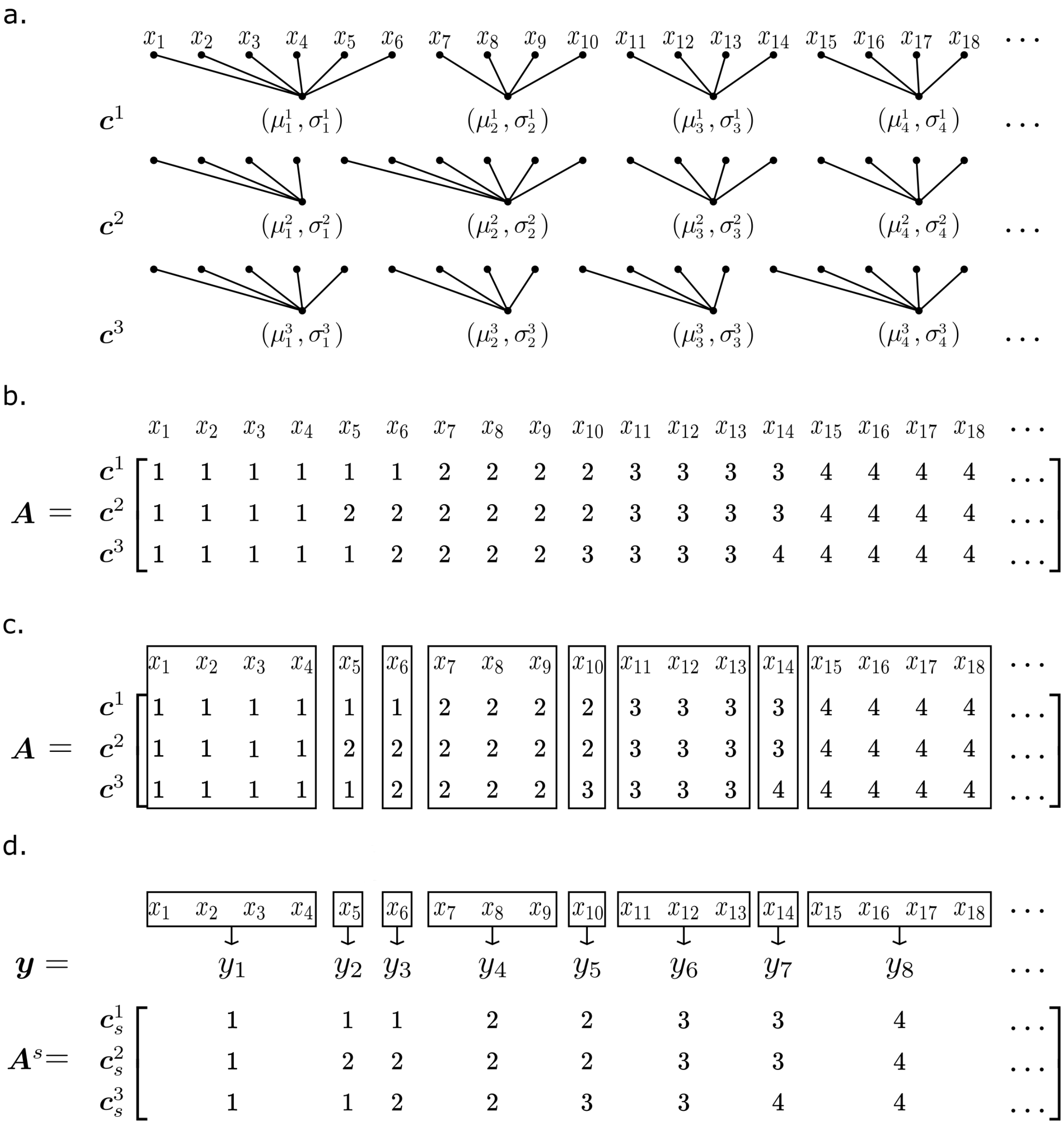

Fig. S5: **Toy example of alignments for junction squiggle, junction squiggle segmentation, and alignments for segmented junction squiggle** a: Example of alignments between a junction squiggle $\boldsymbol{x} = (x_1, x_2, \ldots)$ and 3 candidate squiggles $\boldsymbol{c}^1, \boldsymbol{c}^2, \boldsymbol{c}^3$. b: The alignments are represented by a matrix $\boldsymbol{A}$. c: Segments are defined by consecutive current measurements whose columns in $\boldsymbol{A}$ are the same. d: Current measurements in the junction squiggle are summarised into $\boldsymbol{y}$. The candidate squiggles of $\boldsymbol{y}$ are denoted by $\boldsymbol{c}_s^1, \boldsymbol{c}_s^2, \boldsymbol{c}_s^2$ and the matrix $\boldsymbol{A}^s$ represents the alignments between $\boldsymbol{y}$ and the candidate squiggles $\boldsymbol{c}_s^1, \boldsymbol{c}_s^2, \boldsymbol{c}_s^2$; see Supplementary sections 1.6.1 and 1.6.2 for details.

can be estimated by

$$\hat{\sigma}_s = \sqrt{\frac{\sum_{i=1}^{L}(\tilde{y}_i - \mu_i)^2}{L-1}}, \tag{3}$$

where μ_i is the mean of $\tilde{y}_i$ which can be obtained from the "expected current level model" in Tombo.

1.6.2 Alignments for the summary from junction squiggle segments

The M alignments between the junction squiggle $\boldsymbol{x} = (x_1, \ldots, x_K)$ and the M candidate squiggles $\boldsymbol{c}^1, \ldots, \boldsymbol{c}^M$ are represented by an $M \times K$ matrix $\boldsymbol{A} = [a_{mk}]$, where a_{mk} indicates the index of the mean-standard deviation in $\boldsymbol{c}^m$ where x_k is aligned; supplementary Fig. S5 provides a toy example. Removing redundant columns in $\boldsymbol{A}$ results in an $M \times N$ matrix $\boldsymbol{A}_s = [a^s_{mn}]$, where a^s_{mn} indicates the index of the mean-standard deviation in $\boldsymbol{c}^m_s$ where y_n (or n-th segment) is aligned; see supplementary Fig. S5 for an example.

1.6.3 Practical implementation of junction squiggle segmentation

Segments with only a small number of measurements yield less stable summary values. However, NanoSplicer model does not incorporate the uncertainty of the summary, and so these summary values possibly cause less optimal performance of NanoSplicer. Thus, we filter out summary values that are computed by $\leq s$ current measurements - we used $s = 3$ in the analysis of the paper.

As the number of candidates increases, the number of segments will likely increase as well, as each candidate will contain unique mean-standard deviation elements that require further segmentation. This often leads to increasing numbers of segments with $\leq s$ measurements (see supplementary Fig. S6A). Thus, in practice, we also implement a modified procedure that uses a relaxed definition of segments to improve performance. From the M candidate squiggles $\boldsymbol{c}^1, \ldots, \boldsymbol{c}^M$, we first pick one as a baseline, denoted by $\boldsymbol{c}^b$; we chose one from the mapped

splice junction in the analysis of the paper. Then, we construct candidate-specific segments for each candidate $\boldsymbol{c}^m$ by applying the original segment definition (see section 2.4.1 in the main text) to only two candidates $\boldsymbol{c}^b$ and $\boldsymbol{c}^m$; see supplementary Fig. S6B for an example. For each candidate $\boldsymbol{c}^m$, we can compute the candidate-specific summary $\boldsymbol{y}^m$ using the candidate-specific segments. Then, we approximate the assignment probability in equation (3) of the main text using candidate-specific summaries, $\boldsymbol{y}^1, \ldots, \boldsymbol{y}^M$. Specifically, equation (3) in the main text can be written as:

$$\mathsf{P}(z = m|\boldsymbol{y}, \Theta) \propto \frac{\mathsf{P}(\boldsymbol{y}|z = m, \boldsymbol{c}_s^m, \boldsymbol{A}_s)}{\mathsf{P}(\boldsymbol{y}|z = b, \boldsymbol{c}_s^b, \boldsymbol{A}_s)} \mathsf{P}(z = m), \tag{4}$$

where $\Theta = (\boldsymbol{c}_s^1, \ldots, \boldsymbol{c}_s^M, \boldsymbol{A}_s)$. We then approximate the likelihood ratio $\frac{\mathsf{P}(\boldsymbol{y}|z=m,\boldsymbol{c}_s^m,\boldsymbol{A}_s)}{\mathsf{P}(\boldsymbol{y}|z=b,\boldsymbol{c}_s^b,\boldsymbol{A}_s)}$ using $\frac{\mathsf{P}(\boldsymbol{y}^m|z=m,\boldsymbol{c}_s^m,\boldsymbol{A}_{m,b}^s)}{\mathsf{P}(\boldsymbol{y}^m|z=b,\boldsymbol{c}_s^b,\boldsymbol{A}_{m,b}^s)}$. Here, $\boldsymbol{A}_{m,b}^s$ represents alignments of $\boldsymbol{y}^m$ to $\boldsymbol{c}_s^m$ and $\boldsymbol{c}_s^b$.

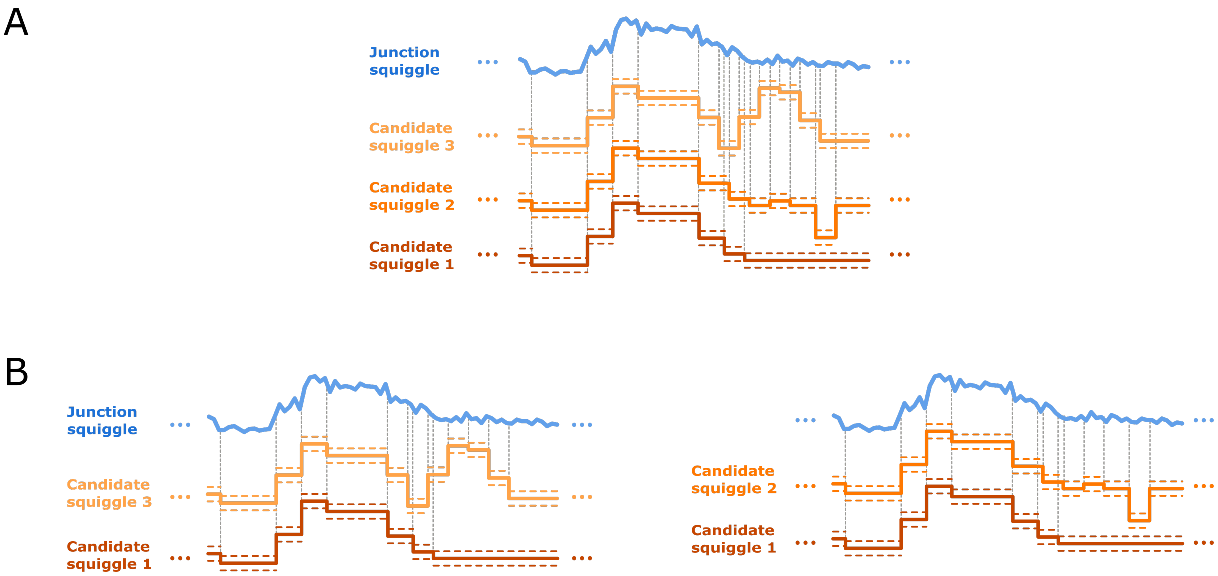

Fig. S6: **Junction squiggle segmentation** A: Example of alignments between a junction squiggle and 3 candidate squiggles. Each current measurement in the junction squiggle (blue) is vertically aligned with its corresponding mean-standard deviation in each candidate squiggle. The black dotted lines are the boundaries of the segments. B: Left and right panels show candidate-specific segments for candidates 3 and 2, respectively (candidate 1 used as a "base candidate").

1.7 Squiggle information quality

The NanoSplicer model assumes that the junction squiggle corresponds to the location of the JWR, however Tombo can potentially align the JWR to the incorrect squiggle location. Therefore, we add a step to filter out junction squiggles that do not have a high quality alignment to any candidate squiggle, suggesting they emanate from an off-target read subsequence. First we measure the alignment quality between a junction squiggle and each of its candidate squiggles using the average log likelihood over the nucleotides of the candidate squiggle. Then, we compute the maximum of these alignment qualities across M candidates, referred to as *squiggle information quality (SIQ)*. In practice, we restrict our splice junction identification to JWRs with SIQ bigger than a threshold to ensure their junction squiggles have high quality alignments at least one of their candidate squiggles. Here, we detail each step to computing SIQ.

Step 1: Measure the alignment quality between a junction squiggle and each of its candidate squiggles. Let $\boldsymbol{x}$ and $\boldsymbol{c}^m$ denote the junction squiggle and the m-th candidate squiggle with length L_m, respectively. We first partition current measurements in $\boldsymbol{x}$ into L_m sections using the alignment between $\boldsymbol{x}$ and $\boldsymbol{c}^m$ (i.e., consecutive measurements aligned to the same mean-standard deviation belong to the same section). Then, we compute the summary $\boldsymbol{y}^m = (y_1^m, \ldots, y_{L_m}^m)$, where y_n^m summarises information in $\boldsymbol{x}$ at its n-th section. Then, we measure the alignment quality between $\boldsymbol{x}$ and $\boldsymbol{c}^m$ using

$$S_m = \frac{\sum_{n=1}^{L_m} \log f(y_n^m | \mu_i^m, \sigma_i^m)}{L_m}, \tag{5}$$

where f is the probability density function of the modified normal distribution (see supplementary section 1.5), and μ_i^m, σ_i^m are the mean and standard deviation in the candidate squiggle $\boldsymbol{c}_s^m$ aligned to y_n^m (see supplementary section 1.6.1 for details on the candidate squiggle $\boldsymbol{c}_s^m$ of the summary values).

Step 2: Compute the maximum of the alignment qualities across M candidates. Step 1

provides $S_1, \ldots, S_M$ that measure the alignment quality between the junction squiggle and the M candidate squiggles. Then, we define squiggle information quality (SIQ) by

$$\text{SIQ} = \max\{S_1, \ldots, S_M\}, \tag{6}$$

and we restrict our splice junction identification to JWRs with SIQ bigger than a threshold to ensure their junction squiggles have high quality alignments at least one of their candidate squiggles.

1.8 Prior mixing proportion as a function of nucleotide composition near splice sites

It has been reported that among the canonical Eukaryotic splice junctions (i.e. "GT-AG" splice junctions), "GTC" or "GTT" is less frequent than "GTA" or "GTG" as intron patterns right after 5' splice sites; "AAG" or "GAG" is less frequent than "CAG" or "TAG" as the 3 nucleotides proceeding 3' splice sites (Irimia and Roy, 2008). For example, it's been reported that the relative frequency of "GTA" or "GTG" at the 5' splice site is larger than 94% in human, mouse, *S. cervisiae* and *S. pombe* (Ast, 2004). Also, our empirical analysis of GENCODE Comprehensive gene annotation (v39) (Frankish *et al.*, 2021) shows that the relative frequency of "GTA" or "GTG" at the 5' splice site and the relative frequency of "CAG" or "TAG" at the 3' splice site are 93.0% and 92.0%, respectively. Minimap2 (Li, 2018) provides an option to exploit these different frequencies of nucleotide composition near splice sites. NanoSplicer also enables users to exploit that information by modelling the prior mixing proportion as a function of nucleotide composition near splice sites.

In our biological RNA data analysis (see section 4 in the main text), we incorporated this information as follows. Let "GT^+" and "GT^-" denote the more frequent (i.e."GTA" or "GTG") and less frequent (i.e. "GTC" or "GTT") intron patterns at 5' splice sites, respectively; similarly, "AG^+" and "AG^-" denote the more and less frequent ones at 3' splice sites. Then, we model

the mixing proportion as

$$\mathsf{P}(Z=m) \propto \begin{cases} h & \text{if the } m\text{-th candidate junction is non-canonical,} \\ 1 & \text{if the } m\text{-th candidate junction has ``GT}^{-}\text{-AG}^{-}\text{'',} \\ r & \text{if the } m\text{-th candidate junction has ``GT}^{+}\text{-AG}^{-}\text{'' or ``GT}^{-}\text{-AG}^{+}\text{'',} \\ r^2 & \text{if the } m\text{-th candidate junction has ``GT}^{+}\text{-AG}^{+}\text{''.} \end{cases} \tag{7}$$

We used $r = 9$ in our biological RNA data analysis, corresponding to a priori assumption that 1) GT^+ at the 5' site and AG^+ at the 3' site are nine times more likely to be splice sites than GT^- and AG^-, respectively; and 2) these propensities at the 5' and 3' splice sites are independent; see supplementary section 2.10.3 for NanoSplicer performance assessments for difference choices of r. We used $h = 1$ in the biological RNA data analysis, a priori assuming that non-canonical junctions have the same propensity to be a splice junction as canonical ones with "GT^--AG^-".

The values for these hyperparameters (h and r) or the function for the prior mixing proportions can be modified for different datasets (e.g., datasets from different organisms) based on prior knowledge on the general propensity of a candidate to be a splice junction. Ultimately, methods that learn the prior mixing proportions from the data at hand may provide a better performance (left for future work).

1.9 Prior mixing proportion when annotated splice junctions are included as candidates

Methods such as FLAIR (Tang *et al.*, 2020) require a set of splice junctions, either from annotations or from matched short reads, to be provided to guide their corrections. While NanoSplicer does not require those input splice junctions, it provides the option to utilise them, for example, in organisms where comprehensive annotations are available. In our NanoSplicer applications with the option to use the human GENCODE v39 annotations (see sections 4.3 and 4.4 in the main text and supplementary section 2.17.2), we considered the following two types of *annotated splice junction* defined by using the information in the human GENCODE v39 annotations and included them as candidates:

- Type 1 annotated splice junction in NanoSplicer: splice junction that appears in annotated transcripts in the human GENCODE v39 annotations
- Type 2 annotated splice junction in NanoSplicer: splice junction that is not a type 1 annotated splice junction, but consists of splice sites appearing in annotated transcripts in the human GENCODE v39 annotations (see Fig. S7 for examples)

Then, we model the mixing proportion as

$$\mathsf{P}(Z = m) \propto q \quad \text{if the } m\text{-th candidate junction is a type 1 annotated splice junction,} \tag{8}$$

and, otherwise,

$$\mathsf{P}(Z = m) \propto \begin{cases} h & \text{if the } m\text{-th candidate junction is non-canonical,} \\ 1 & \text{if the } m\text{-th candidate junction has ``GT}^-\text{-AG}^-\text{'',} \\ r & \text{if the } m\text{-th candidate junction has ``GT}^+\text{-AG}^-\text{'' or ``GT}^-\text{-AG}^+\text{'',} \\ r^2 & \text{if the } m\text{-th candidate junction has ``GT}^+\text{-AG}^+\text{''.} \end{cases} \tag{9}$$

We used $q = 81$ in our analysis (sections 4.3 and 4.4 in the main text and supplementary section 2.17.2), giving the same weight to type 1 annotated splice junctions and "GT^+-AG^+" junctions (as we used $r^2 = 81$ in the analysis). Thus, we gave a higher weight for junctions appearing in the annotated transcripts (type 1), but treated type 2 annotated splice junctions as other candidates (whose weights are determined by nucleotide compositions near splice sites).

2 Supplementary result

2.1 Data description

Synthetic RNA nanopore sequencing dataset: The synthetic sequins dataset was generated by Dong *et al.* (2021). The original dataset (GSE151984) contains duplicate libraries of each sequin mix (A and B), for a total of 4 cDNA libraries. The two sequin mixes contain the

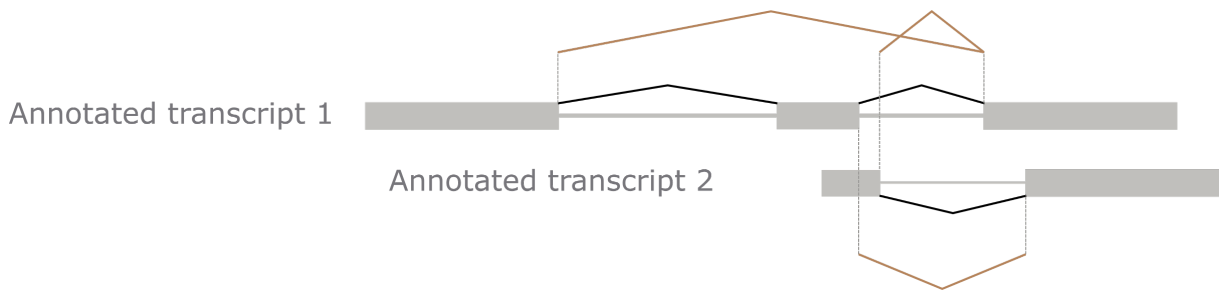

Fig. S7: **Annotated splice junctions in NanoSplicer.** Grey boxes connected by grey lines represent annotated transcripts provided as input. Black lines indicate type 1 annotated splice junctions that appear in the annotated transcripts and brown lines indicates type 2 annotated splice junctions that consist of splice sites appearing in the annotated transcripts.

same transcripts but at differing concentrations. Libraries were constructed with the cDNA-PCR (SQK-PCS109) sequencing kit and PCR Barcoding (SQK-PBK004) kit using the standard protocol. The final pooled library was sequenced on a R9.4.1 MinION flow cell. In our analysis, we only used reads from one of the sequin mix A libraries. The *fast5* files containing the raw nanopore squiggles are available from the European Nucleotide Archive (ENA) under accession ERS7273757.

Biological short-read sequencing dataset: We used the dataset generated by Holik *et al.* (2017). The original dataset contains 40 samples from two lung cancer cell lines (NCI-H1975 and HCC827). We utilised three of the samples (Accession: GSM1564311, GSM1564316 and GSM1564326) from the NCI-H1975 cell line that was prepared using the Illumina TruSeq RNA v2 kit (Poly-A mRNA) followed by sequencing on an Illumina HiSeq 2500.

Biological RNA nanopore sequencing dataset: To assess the performance of NanoSplicer, we used a subset of a dataset generated by Matthew Ritchie's Lab at the Walter and Eliza Hall Institute of Medical Research, Australia. The original dataset contains 6 libraries from two lung cancer cell lines (NCI-H1975 and HCC827). Libraries were constructed with the cDNA-PCR (SQK-PCS109) sequencing and PCR Barcoding (SQK-PBK004) kits using the standard protocol. The final libraries were sequenced on a PromethION flow cell (FLO-PRO002). We

utilised a portion of the reads mapping to chromosome 1 from one of the NCI-H1975 libraries. The squiggles (*fast5* files) of the reads included in our data analysis are available from ENA (Accession: ERS7273453).

2.2 Nanopore data : Basecalling of raw signals (squiggles)

We basecalled squiggles (*fast5* files) described in supplementary section 2.1 using Guppy 3.6.1. When running Guppy, we used configuration files *dna_r9.4.1_450bps_hac.cfg* for the synthetic RNA dataset and *dna_r9.4.1_450bps_hac_prom.cfg* for biological RNA dataset to get *fastq* files. Using Guppy, we filtered out low quality reads and demultiplex barcoded reads and trimmed adaptor sequences with the following command line parameters: *"--calib_detect --qscore_filtering --barcode_kits "SQK-PBK004" --trim_barcodes"*.

2.3 Nanopore data : Mapping of basecalled reads

2.3.1 Mapping nanopore reads to a reference genome (splice-aware)

NanoSplicer requires mapped nanopore reads as input. We used minimap2 to map nanopore reads to a reference genome (both synthetic and biological data).

Synthetic RNA nanopore reads: Using minimap2 (version 2.17-r943-dirty), we mapped the synthetic RNA reads to the sequin genome v2.4 with the following set of parameters: *"-ax splice -t 32 -k 15 -ub --eqx --secondary=no --sam-hit-only --splice-flank=no"*. We activated *"--eqx"* option in minimap2 to output the CIGAR string with mismatch information. By default, the CIGAR string in the output BAM file uses "M" to indicate a match or mismatch. With *"--eqx"*, the matches and mismatches are denoted as "=" and "X", respectively. We used the mismatch information when calculating the junction alignment qualitiy (see supplementary section 2.6). We deactivated the "splice flank" sequence preference option (*"--splice-flank=no"*) as this preference is not present in sequins; see supplementary section 1.8 for details on the

intron sequence preference.

Biological RNA nanopore reads: Using minimap2 (verson 2.17-r943-dirty), we mapped the biological RNA reads to the human GRCh38 assembly (`ftp://ftp.ncbi.nlm.nih.gov/genomes/all/GCA/000/001/405/GCA_000001405.15_GRCh38/seqs_for_alignment_pipelines.ucsc_ids/GCA_000001405.15_GRCh38_no_alt_analysis_set.fna.gz`, which is the optimal GRCh38 version for minimap2 (Li, 2017)). We ran minimap2 with the following set of parameters: *"-ax splice -t 32 -k 15 -ub --eqx --secondary=no --sam-hit-only"*. The setting is identical to what we used in the synthetic RNA data above, except for the removal of *" --splice-flank=no"*.

2.3.2 Mapping (synthetic) nanopore reads to the sequin isoforms

In our synthetic data analysis (section 3 in the main text), we defined a ground truth splice junction for each JWR by choosing one among the known 745 sequin splice junctions. To identify a 1-1 correspondence between each read and a sequin isoform, we mapped the reads to the sequin isoforms using minimap2. We ran minimap2 with command *"minimap2 -ax map-ont -t 32 --secondary=no --sam-hit-only"*, which is the setting for mapping nanopore reads to a reference without splicing. With the parameter "--secondary=no", the resulting BAM file did not contain any secondary alignments. Furthermore, to remove the potential ambiguity, we filtered out the reads with supplementary alignment(s) to a different isoform.

2.4 Short read data: pre-processing, mapping, and identifying splice junctions supported by the mapped short reads

In short-read sequencing data, base qualities near the ends of reads tend to be poor. All leading and trailing bases below a Phred quality score of 20 were removed using Trimmomatic v0.36 (Bolger *et al.*, 2014). We mapped the short reads to GRCh38 using STAR v2.7.3a (Dobin *et al.*, 2013) with the following parameters: "--outSAMtype BAM SortedByCoordi-

nate --twopassMode Basic". To improve the mapping quality, we provided the GENCODE Comprehensive gene annotation (v39) (Frankish *et al.*, 2021) to guide the mapping. We used pysam v0.15.2 (`https://github.com/pysam-developers/pysam`) to find splice junctions supported by the mapped short reads, as well as the number of reads mapped to each of them.

2.5 Comparing splice junctions supported by short and long-reads mapping

In the biological RNA data analysis in section 4 of the main text, we used short reads to define a ground truth for each JWR – treating a splice junction with at least 3 mapped short reads within 10 bases of the mapped splice sites as a ground truth for that JWR. Thus, in this section, we compared the number of unique splice junctions identified by both short and long-read mapping and their overlap.

We first identified splice junctions from the short- and long-read mapping results (see supplementary sections 2.3 and 2.4 for details of the long- and short-read mapping, respectively). Then, we found 22,383 unique splice junctions with at least 3 mapped short reads (equivalent to the minimum number of mapped short reads for the ground truths in our analysis) and 53,522 unique splice junctions supported by mapped long reads (no minimum number of mapped long reads required); see Fig. S8A.

Among the 53,522 unique splice junctions identified by long reads, 16,138 were confirmed by short-read junctions (i.e., supported by at least 3 mapped short reads) and and 37,384 were not; see Fig. S8A. Almost all the 37,384 junctions found only in long reads were supported by only a few long reads - 78% (29,063) were supported by only 1 long-read and 95% (35,172) were supported by < 5 long-reads; see Fig. S8B. Moreover, these 37,384 JWRs had lower junction alignment qualities (JAQs) than those confirmed by short reads (Fig. S8C), suggesting

many were potential mapping errors. Therefore, many of these long-read only junctions were expected to be candidates for correction with Nanosplicer. This was indeed the case, with the number of long-read only junctions decreasing by 12,452 after NanoSplicer correction (from 37,384 to 24,932; see Fig. S8A). In contrast, almost all (98.8%) of long-read junctions confirmed by short-read were retained after Nanosplicer correction. Note that our overall results encompass these long-read only junctions (after NanoSplicer correction), which are part of the 2.9% Nanosplicer error rate we reported in Table 1. As detailed in supplementary section 2.11, most Nanosplicer errors and hence long-read only junctions are "completely missed JWRs" where the initial mapping placed the JWR far from the true splice junction and hence NanoSplicer was unable to include the true junction as a candidate.

Performing the opposite comparison, among the 22,383 unique splice junctions identified by short reads, 16,138 were confirmed by long-read junctions and 6,245 were not; see Fig. S8A. The 6,245 junctions found only in short reads tend to be supported by a low number of short-reads (Fig. S8D), suggesting they are low-abundance junctions, which are most likely not identified in long-reads due to differences in sequencing depth.

2.6 Junction alignment quality (JAQ)

Basecalling errors can result in low quality alignments between the JWR and the reference genome. Therefore we hypothesized that the initial mapping would perform poorly for JWRs with high basecalling errors and that the advantage of NanoSplicer will be greatest for these. To test this, we adapted the definition of read accuracy in Rang *et al.* (2018) and defined a *junction alignment quality* (JAQ) by

$$\text{JAQ} := \frac{\sum(\text{matched bases in an alignment})}{\sum(\text{matched/mismatched/inserted/deleted bases in an alignment})}. \tag{10}$$

We obtained the number of matched, mismatched, inserted or deleted bases from the CIGAR string in the mapping result (SAM/BAM file). In the CIGAR string from splice-aware mappers,

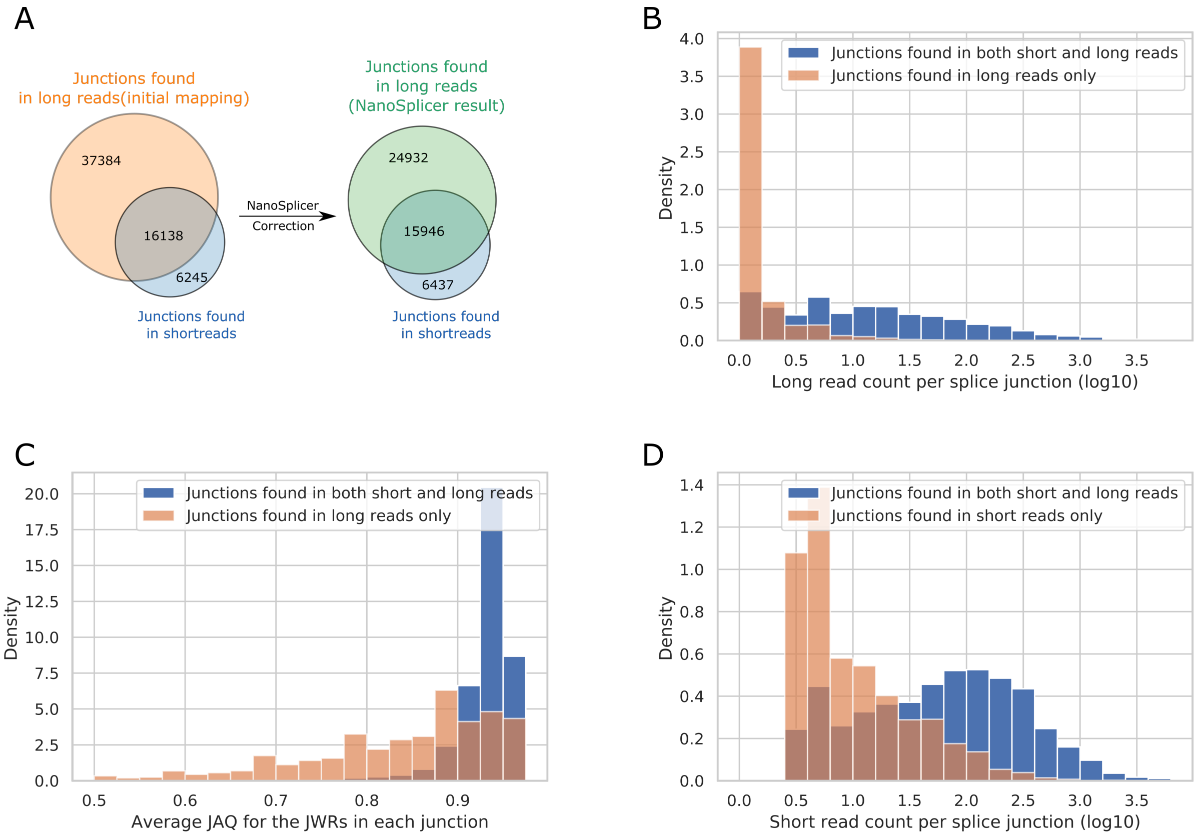

Fig. S8: **Splice junctions supported by short and long-reads mapping** Panel A shows number of unique splice junctions found in long and short reads and their overlap, before and after NanoSplicer correction. For 16,138 unique splice junctions identified by both short and long reads (blue) and 37,384 unique splice junctions supported only by long reads (orange), panel B shows distributions of long-read count for each junction (i.e., the number of long reads mapped to each junction) and panel C shows distributions of average JAQ for each junction. The average JAQ for each unique splice junction was calculated by averaging JAQs from all JWRs mapped to that unique splice junction. For the 16,138 unique splice junctions identified by both short and long reads (blue) and 6,245 unique splice junctions supported only by short reads (orange), panel D shows distributions of short read count for each junction. Note that junctions supported by long reads and long-read counts in B-D are based on the initial mapping results (before NanoSplicer correction).

the label for reference skipping (i.e. the mapped introns) is different from the deletions, and so the reference skipping is not counted in either numerator or denominator in equation 10. Only the mismatches, insertions or deletions near the mapped splice sites are counted. In the analysis of the paper, we used 25 bases (including mismatched, inserted and deleted bases) preceding the mapped 5' splice site and 25 bases after the mapped 3' splice site as bases in an alignment to compute JAQ.

2.7 Non-analysable JWRs

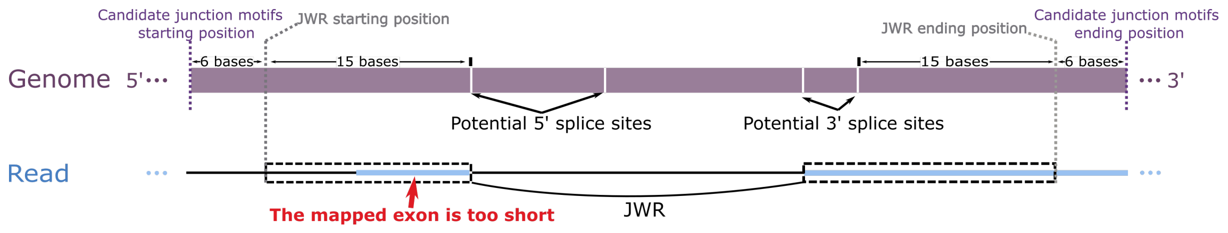

Fig. S9: **NanoSplicer fails to obtain candidate junction motifs when the mapped exon is too short.** NanoSplicer can not construct candidate junction motifs if the mapped exons do not overhang candidate junction motif start/end positions.

NanoSplicer failed to run on 0.49% of all JWRs in the synthetic dataset and 0.26% in the biological dataset due to the following two reasons.

1. 0.41% and 0.21% of the total JWRs in the synthetic and biological datasets, respectively: Mapped exons were too short, so NanoSplicer could not construct candidate junction motifs (supplementary Fig. S9). These JWRs are potentially related to spurious splice junction mapping. Only 20.5% and 58.4% of these JWRs (the synthetic and biological datasets, respectively) are initially mapped to true splice junctions.

2. 0.08% and 0.05% of the total JWRs in the synthetic and biological datasets, respectively: Tombo failed to align squiggles to reads, so NanoSplicer could not locate junction squiggles. These JWRs may have low squiggle quality. 86.2% and 93.4% of these JWRs (the

synthetic and biological datasets, respectively) are initially mapped to true splice junctions. The initial mapping accuracy for these JWRs are lower than that for JWRs for which we successfully ran NanoSplicer (92.2% and 95.7% for the synthetic and biological datasets, respectively).

Note that these JWRs were omitted when we reported the number of JWRs in NanoSplicer output and calculated the accuracy of the NanoSplicer in the main text and all the following supplementary sections.

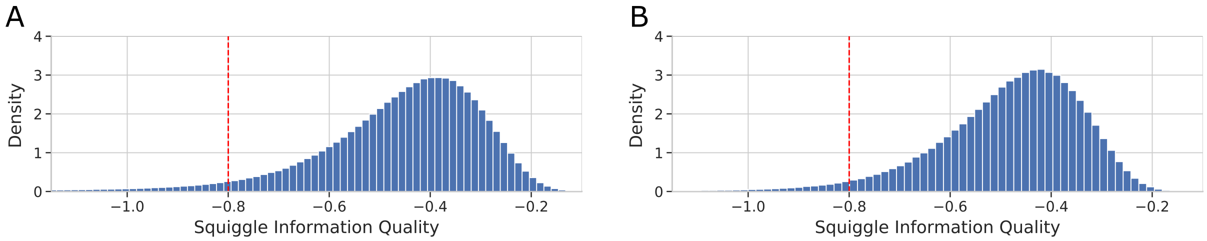

Fig. S10: **Empirical distributions of SIQ.** To choose a suitable threshold for SIQ, we first computed SIQ for all analysable JWRs (see Supplementary section 2.7 for more details of non-analysable JWRs) included in our analysis (3,509,611 and 1,875,139 JWRs for synthetic and biological data, respectively), and construct an empirical distribution of SIQ by pooling all SIQ values (A and B panels for synthetic and biological data, respectively). Then, assuming that most JWRs are well aligned to correct squiggle locations, we choose -0.8 as a SIQ threshold that identifies junction squiggles whose SIQ values much smaller than a majority of SIQs in the distribution (5.4% and 4.5% of all JWRs $<$ -0.8 for synthetic and biological data, respectively). In supplementary section S2.5.1, we assess NanoSplicer performance for different choices of a SIQ threshold and discuss how the choice of thresholds involves trade-offs between accuracy and the ability to identify splice junctions.

2.8 Contribution of SIQ, assignment probability and NanoSplicer correction to the accuracy improvement

In our analysis in section 3 [section 4] in the main text, NanoSplicer identified splice junctions for 3,066,881 JWRs [1,724,883 JWRs] whose SIQ $>$ -0.8 and strongest assignment probability $>$ 0.8; supplementary Fig. S10 shows empirical distributions of SIQ. So the accuracy of NanoSplicer is evaluated on this smaller set while we used all JWRs (3,526,941 JWRs [1,880,011

	Junction alignment quality	All JWRs	JWRs with SIQ>-0.8	JWRs with SIQ>-0.8 & strongest assginment probability >0.8
Synthetic RNA dataset	0-0.8	282,332 (111,212)	181,712 (38,706)	154,033 (30,574)
	0.8-0.85	201,933 (20,803)	179,710 (12,408)	158,601 (10,456)
	0.85-0.9	604,024 (27,959)	571,291 (20,069)	516,159 (17,206)
	0.9-0.95	739,472 (16,984)	716,814 (12,865)	661,612 (11,166)
	0.95-1	1,699,180 (16,096)	1,669,955 (14,268)	1,576,476 (13,295)
Biological RNA dataset	0-0.8	98,467 (16,821)	72,451 (66,99)	66,090 (5,346)
	0.8-0.85	95,238 (4,069)	85,179 (27,92)	79,773 (2,335)
	0.85-0.9	320,238 (8,874)	301,899 (71,64)	286,979 (6,304)
	0.9-0.95	412,499 (8,415)	398,800 (75,14)	383,404 (6,902)
	0.95-1	953,569 (13,990)	933,238 (13,333)	908,637 (12,717)

Table S1: **Number of JWRs in each junction alignment quality (JAQ) bin.** The table presents the number of JWRs in each junction alignment quality (JAQ) bin for all JWRs, JWRs after SIQ filtering, and JWRs after SIQ and strongest assignment probability filtering. The numbers in the bracket represent the number of “completely missed JWRs”

JWRs]) to compute the accuracy of initial mapping. Thus, multiple factors could contribute to the increased accuracy of NanoSplicer. These include the ability of SIQ and assignment probability to identify wrongly mapped JWRs, as well as NanoSplicer correction of the initial mapping results. To investigate the contributions of these factors, we compute the accuracy of the initial mapping on two sets of JWRs: 3,319,482 JWRs [1,791,567 JWRs] after SIQ filtering (SIQ $>$ -0.8) and 3,066,881 JWRs [1,724,883 JWRs] after SIQ and strongest assignment probability filtering (SIQ $>$ -0.8 and strongest assignment probability $>$ 0.8). Note the the non-analysable JWRs (see supplementary section 2.7) were also filtered out when applying SIQ and/or assignment probability filtering. Supplementary Fig. S11 shows the accuracy for JWRs with different ranges of junction alignment quality. Supplementary Table S1 shows the number of JWRs in each junction alignment quality bin for all JWRs, JWRs after SIQ filtering, and JWRs after SIQ and strongest assignment probability filtering, including completely missed JWRs. Supplementary Table S2 shows more detailed impact of NanoSplicer correction: the number of JWRs which were incorrectly mapped and then corrected by NanoSplicer, as well as JWRs which were correct in the initial mapping but wrongly identified by NanoSplicer.

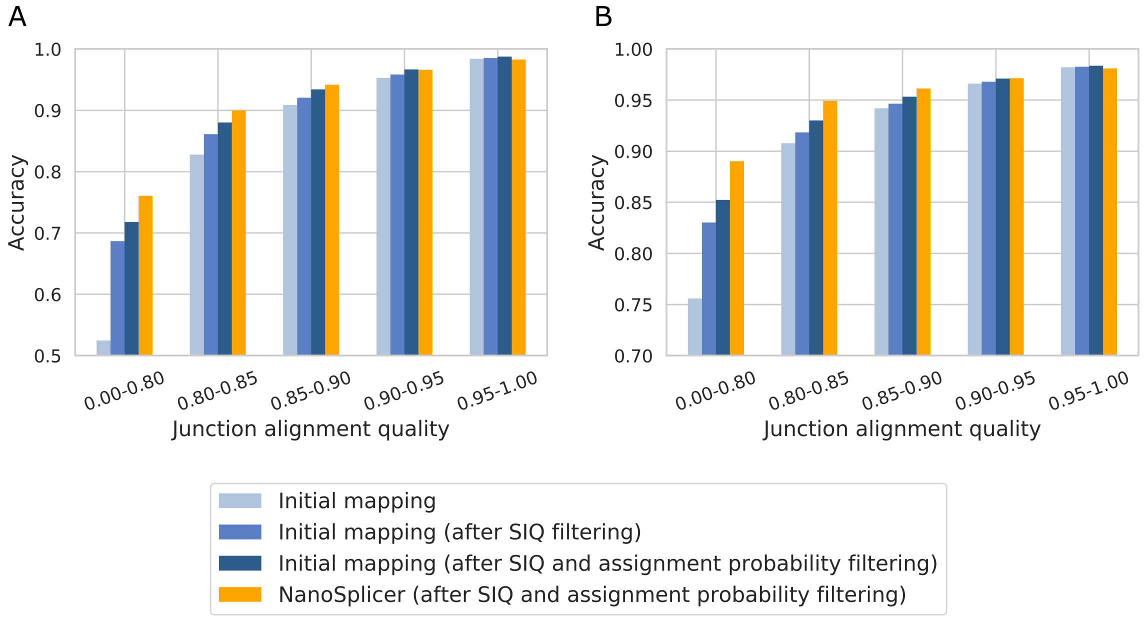

Fig. S11: **Contribution of SIQ, assignment probability and NanoSplicer correction to the accuracy improvement.** A: Accuracy of NanoSplicer and the initial mapping in the synthetic RNA dataset. B: Accuracy of NanoSplicer and the initial mapping in the biological RNA dataset.

	Junction alignment quality	JWRs with SIQ>-0.8 & strongest assignment probability >0.8	Incorrectly mapped JWRs (Number and proportion of JWRs corrected by NanoSplicer)	Correctly mapped JWRs (Number and proportion of JWRs wrongly identified by NanoSplicer
Synthetic RNA dataset	0-0.8	154,033	43,402 (10,491, 24.2%)	110,631 (3,944, 3.6%)
	0.8-0.85	158,601	18,977 (6,693, 35.3%)	139,624 (3,583, 2.6%)
	0.85-0.9	516,159	33,852 (12,442 , 36.8%)	482,307 (8,653 , 1.8%)
	0.9-0.95	661,612	21,960 (7,308, 33.3%)	639,652 (7,724 , 1.2%)
	0.95-1	1,576,476	19,161 (3,266, 17.0%)	1,557,315 (10,993 ,0.7%)
Biological RNA dataset	0-0.8	66,090	9,764 (3,557, 36.4%)	56,326 (1,051, 1.9%)
	0.8-0.85	79,773	5,584 (2,499, 44.8%)	74,189 (973,1.3%)
	0.85-0.9	286,979	13,435 (4,873 ,36.3%)	273,544 (2,538, 1.0%)
	0.9-0.95	383,404	111,33 (2,666, 23.4%)	372,271 (2,520, 0.7%)
	0.95-1	908,637	14,985 (1,066, 7.1%)	893,652 (3,428, 0.04%

Table S2: **Contribution of NanoSplicer correction to the initial mapping result.** The table presents, for each junction alignment quality (JAQ) bin, the number of JWRs after SIQ and strongest assignment probability filtering, and among them, the number of JWRs which were incorrectly mapped and then corrected by NanoSplicer, as well as JWRs which were correct in the initial mapping but wrongly identified by NanoSplicer.

	Junction alignment quality	JWRs with SIQ>-0.8 (out of all JWRs)	JWRs with strongest assignment probability >0.8 (out of all JWRs with SIQ>-0.8)	JWRs with SIQ>-0.8 & strongest assignment probability >0.8 (out of all JWRs)
Synthetic RNA dataset	0-0.8	64.36%(34.80%)	84.77%(78.99%)	54.56%(27.49%)
	0.8-0.85	88.99%(59.65%)	88.25%(84.27%)	78.54%(50.26%)
	0.85-0.9	94.58%(71.78%)	90.35%(85.73%)	85.45%(61.54%)
	0.9-0.95	96.94%(75.75%)	92.30%(86.79%)	89.47%(65.74%)
	0.95-1	98.28%(88.64%)	94.40%(93.18%)*	92.78%(82.60%)
Biological RNA dataset	0-0.8	73.58%(39.83%)	91.22%(79.80%)	67.12%(31.78%)
	0.8-0.85	89.44%(68.62%)	93.65%(83.63%)	83.76%(57.39%)
	0.85-0.9	94.27%(80.73%)	95.06%(88.00%)	89.61%(71.04%)
	0.9-0.95	96.68%(89.29%)	96.14%(91.86%)	92.95%(82.02%)
	0.95-1	97.87%(95.30%)	97.36%(95.38%)	95.29%(90.90%)

Table S3: **Proportions of JWRs and completely missed JWRs after SIQ and/or assignment probability filtering.** This table shows the proportions of JWRs after SIQ filtering (first column) and JWRs after SIQ and assignment probability filtering (among all JWRs or JWRs who pass SIQ filtering; the third and second columns). The numbers in brackets are those proportions for "completely missed JWRs".

2.9 JWRs filtered by SIQ and/or assignment probability are enriched in completely missed JWRs

Completely missed JWRs are JWRs whose mapped splice junctions are far away (> 10 nucleotides away in the analysis of the paper) from any true splice junctions, so their true splice junctions are not included as candidate junctions. Here, we assess how well SIQ and/or assignment probability in NanoSplicer recognise and filter out these JWRs. Supplementary Table S3 shows the proportions of JWRs and completely missed JWRs after SIQ and/or assignment probability filtering (these proportions have been computed from the numbers in supplementary Table S1). For each proportion, we performed the hypothesis testing for $H_0 : p_1 = p_2$ versus $H_1 : p_1 < p_2$, where p_1 is the proportion of completely missed JWRs after filtering and p_2 is the proportion of non-completely missed JWRs after filtering. For most cases, the SIQ and/or assignment probability filtered out more JWRs for completely missed JWRs than non-completely missed JWRs (p-value $< 10^{-15}$ for all cases except for the case with "*" in supplementary Table S3 which has p-value $< 10^{-9}$), supporting that SIQ and/or assignment probability filtering help identify JWRs without true junctions as candidates.

2.10 Assessing NanoSplicer performance for different choices of SIQ and strongest assignment probability thresholds, quantiles in the modified normal distribution, and a hyperparameter in the mixing proportions

In our analysis in the paper, we chose -0.8 as a SIQ threshold, 0.8 as a strongest assignment probability threshold (sections 3 and 4 in the main text), $\alpha = 0.01$ for quantiles in the modified normal distributions (supplementary section 1.5), and $r = 9$ as a hyperparameter in the mixing proportions (supplementary section 1.8). Here we assess NanoSplicer performance for different choices of those values using randomly selected 100K JWRs from all analysable JWRs in the synthetic data and biological data (biological data has been used for the hyperparameter in the mixing proportions, see Supplementary section 2.7 for the details of the tiny proportion of non-analysable JWRs).

2.10.1 Squiggle information quality (SIQ) and strongest assignment probability threshold

We used randomly selected 100K JWRs from the synthetic data to assess NanoSplicer performance for different choices of SIQ and strongest assignment probability thresholds. Supplementary Fig. S12 C & D show accuracy of NanoSplicer and the initial mapping for varying SIQ threshold values (-0.9, -0.8, -0.7, -0.6) at a single strongest assignment probability threshold (0.8) and for varying strongest assignment probability threshold values (0.7, 0.8, 0.9) at a single SIQ threshold (-0.8), respectively. The NanoSplicer performance is robust to difference choices of the thresholds. More stringent threshold values (larger threshold values) lead to higher accuracy, especially for JWRs with low junction alignment quality (JAQ). However, more stringent threshold values reduce the number of JWRs for which NanoSplicer is able to identify splice junctions (see Supplementary Fig. S12 A & B). For example, 89.5% of JWRs with JAQ $\leq$ 0.9 have SIQ $>$ -0.9, while only 57.5% of these JWRs have SIQ $>$ -0.6.

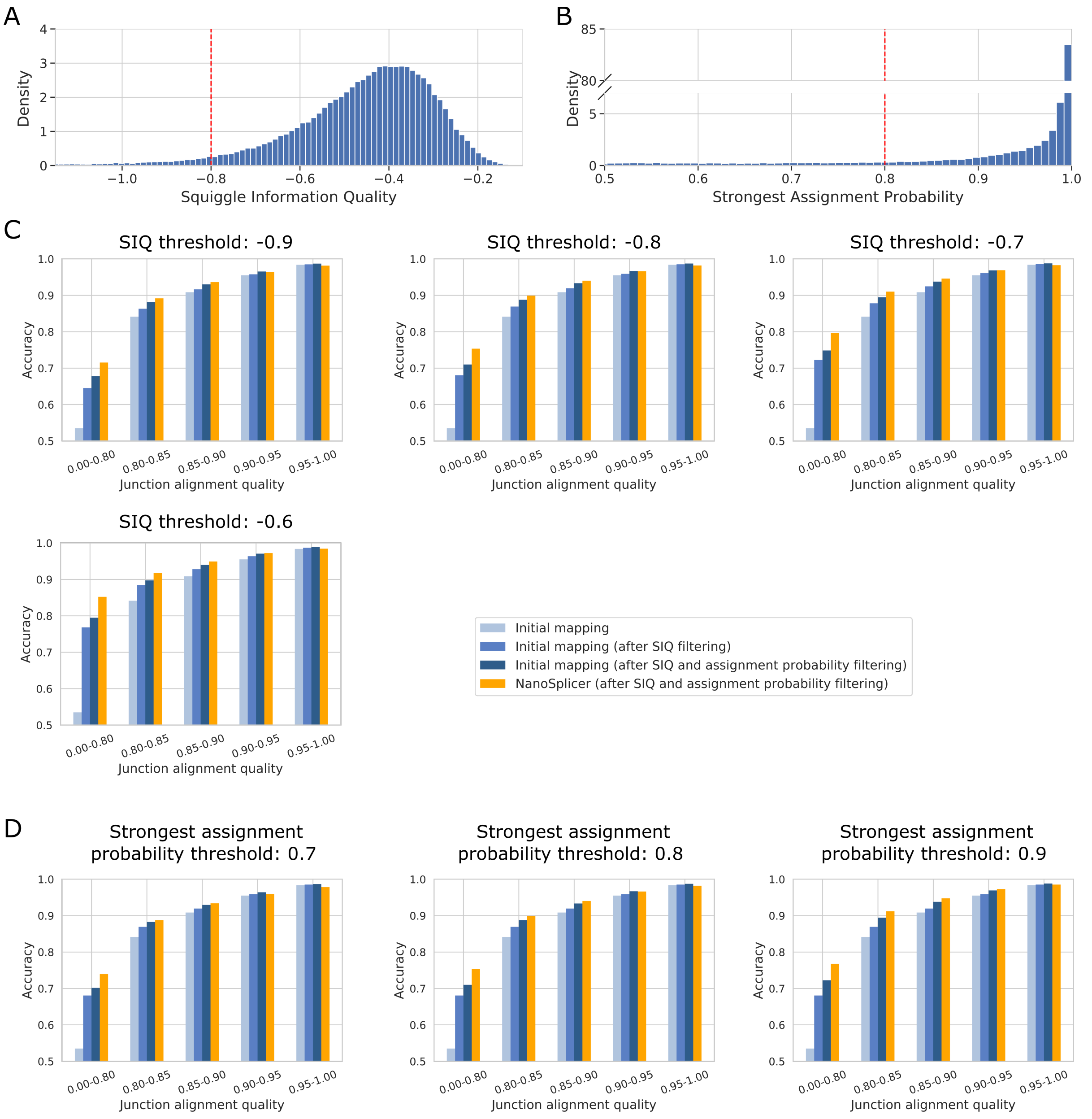

Fig. S12: **Assessment of NanoSplicer performance for different choices of SIQ and strongest assignment probability thresholds.** Distributions of SIQ values (A) and strongest assignment probabilities (B) from randomly selected 100K JWRs from the synthetic data. The red dashed lines indicate the threshold values used in our analysis of the main text. C: Accuracy of NanoSplicer and the initial mapping for varying SIQ threshold values (-0.9, -0.8, -0.7, -0.6) at a single strongest assignment probability threshold (0.8). D: Accuracy of NanoSplicer and the initial mapping for varying strongest assignment probability threshold values (0.7, 0.8, 0.9) at a single SIQ threshold (-0.8).

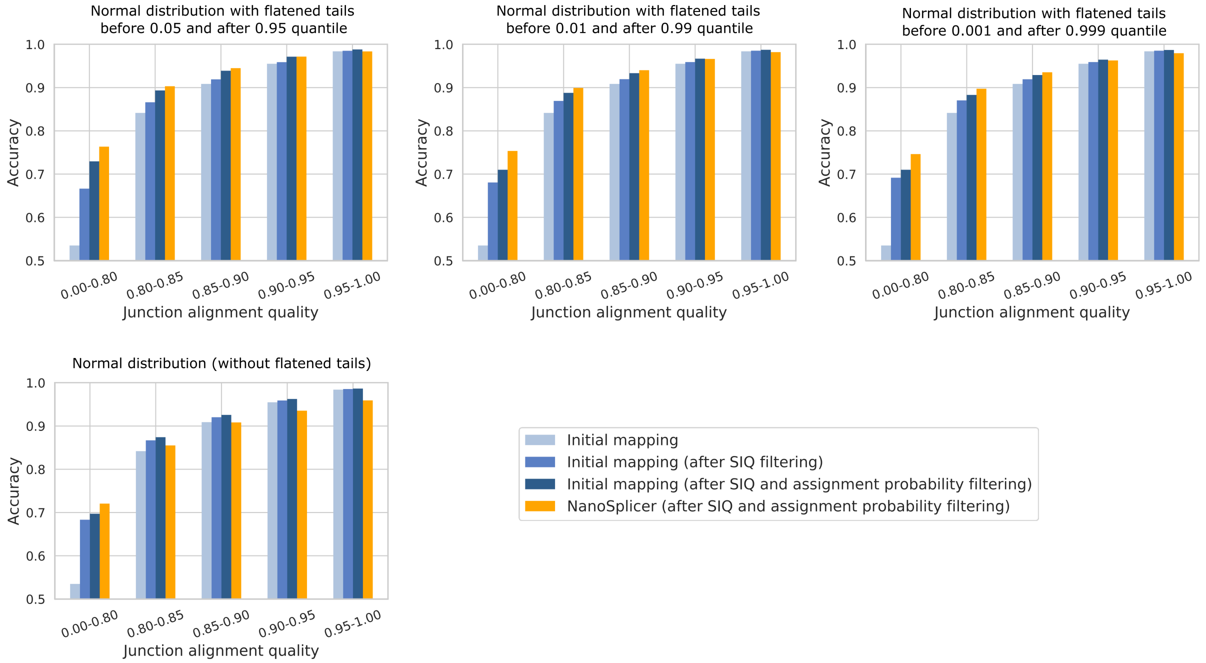

Fig. S13: **Assessment of NanoSplicer performance for different choices of α.** The α has been used to define quantiles in the modified normal distributions.

2.10.2 α for quantiles in the modified normal distributions

Our NanoSplicer model (section 2.4.2 in the main text) uses the modified normal distributions which have flat tails, making our methods robust to measurements that match none of the M candidate squiggles. Supplementary section 1.5 introduces details of these distributions which have α as a parameter to define q_α and $q_{1-\alpha}$ in the distributions. Here, we used the randomly selected 100K JWRs from the synthetic data to assess NanoSplicer performance for different choices of α. Supplementary Fig. S13 show the accuracy of NanoSplicer and the initial mapping for varying α values (0.05, 0.01, 0.001, 0). Note that the modified normal distributions with $\alpha = 0$ are equivalent to normal distributions. The use of the modified normal distributions ($\alpha > 0$) helps improve NanoSplicer correction of the initial mapping results, particularly for JWRs with JAQ > 0.8. There was no significant difference among the results of $\alpha > 0$.

2.10.3 Hyperparameter r in the mixing proportions

In our biological RNA data analysis, we used $r = 9$ in the prior mixing proportions, corresponding to a priori assumption that 1) GT^+ at the 5' site and AG^+ at the 3' site are 9 (=r) times more likely to be splice sites than GT^- and AG^-, respectively; and 2) these propensities at the 5' and 3' splice sites are independent (see supplementary section 1.8 for details). Here, we assess NanoSplicer performance for different choices of r using randomly selected 100K JWRs from the biological data. Supplementary Fig. S14 shows accuracy of NanoSplicer and the initial mapping for varying r values between 1 to 96. Note that $r = 1$ corresponds to a priori assumption that nucleotide composition near splice sites is not related to the propensity of a candidate to be a splice junction. Modelling a prior mixing proportion as a function of nucleotide composition near splice sites ($r > 1$) helps improve NanoSplicer performance. NanoSplicer performance is robust to difference values of r - at all values of r between 1 to 98, NanoSplicer accuracy is higher than that of the initial mapping (95.6%).

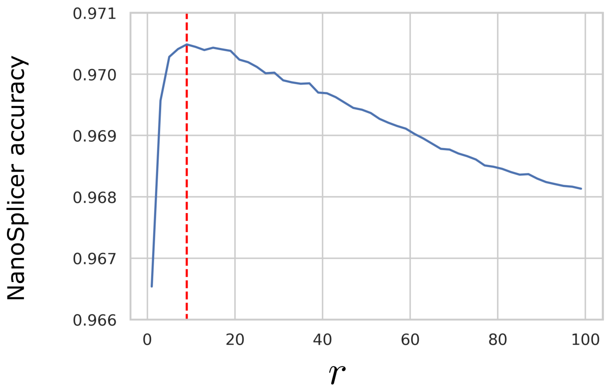

Fig. S14: **Assessment of NanoSplicer performance for different choices of a hyperparameter r in the mixing proportions.** The red dashed line indicates $r = 9$ which has been used in our analysis of the main text.

2.11 JWRs remaining wrong after NanoSplicer correction

Among 3,066,881 JWRs which passed the SIQ and strongest assignment probability filtering in synthetic data analysis [1,724,883 JWRs in biological data analysis], 4.3% [2.9%] of the JWRs still remain wrong after NanoSplicer correction. In this section, we summarise several reasons for these JWRs with NanoSplicer errors.

The majority of these JWRs (66.0% and 66.2% for synthetic and biological data sets, respectively) are completely missed JWRs (supplementary Table S4). Completely missed JWRs occur when the initial mapping is too distant ($>$10 bases) from the true site for it to be included in the list of candidates and all candidates which can be assessed are incorrect. While Nanosplicer filters out many of these completely missed JWRs (see supplementary section 2.9), those not filtered out make up the bulk of the NanoSplicer errors.

In addition to the above, a smaller proportion of the errors are from JWRs whose squiggles do not have enough information to identify the correct splice junctions. While we implement multiple filtering steps (SIQ and strongest assignment probability) to remove most squiggles that fall into this category, there are trade-offs between accuracy and the ability to identify splice junctions. The current analysis used -0.8 and 0.8 as threshold values for the SIQ and strongest assignment probability filtering. We find that 20.2% [21.2%] and 28.0% [23.2%] of the incorrect JWRs in synthetic data analysis [biological data analysis] have SIQ from -0.5 to -0.8 or a strongest assignment probability from 0.8 to 0.99, respectively; see supplementary Table S4. So these JWRs passed our filtering, but with lower levels of confidence in their accuracy. Supplementary Fig. S12 shows that the use of more stringent threshold values leads to higher accuracy by filtering out these JWRs, but at the expense of many correctly identified JWRs.

Finally, 2.2% [3.4%] of the incorrect JWRs have high SIQ ($>$ -0.5) and strongest assignment probability ($>$ 0.99), but NanoSplicer didn't identify the correct splice junctions for them. We observed that NanoSplicer performs poorly on these JWRs because our model does not

Synthetic Data

	SIQ range (strongest assignment probability > 0.8)	Number of JWRs	Proportion	Strongest assignment probability range (SIQ > -0.8)	Number of JWRs	Proportion
Completely missed JWRs	>-0.8	87,024	66.0%	>0.8	87,024	66.0%
Not completely missed JWRs (JWRs within 10 bases of true splice junctions)	-0.8 to -0.75	2,552	1.9%	0.8 to 0.85	8,080	6.1%
	-0.75 to -0.7	3,331	2.5%	0.85 to 0.9	8,702	6.6%
	-0.7 to -0.65	4,144	3.1%	0.9 to 0.95	10,030	7.6%
	-0.65 to -0.6	5,002	3.8%	0.95 to 0.99	10,166	7.7%
	-0.6 to -0.55	5,696	4.3%	>0.99	8,047	6.1%
	-0.55 to -0.5	5,919	4.5%			
	>-0.5	18,381	13.9%			
Total	-	132,049	100%	-	132,049	100%

Biological Data

	SIQ range (strongest assignment probability > 0.8)	Number of JWRs	Proportion	Strongest assignment probability range (SIQ > -0.8)	Number of JWRs	Proportion
Completely missed JWRs	>-0.8	33,604	66.2%	>0.8	33,604	66.2%
Not completely missed JWRs (JWRs within 10 bases of true splice junctions)	-0.8 to -0.75	1,141	2.2%	0.8 to 0.85	2,512	4.9%
	-0.75 to -0.7	1,456	2.9%	0.85 to 0.9	2,706	5.3%
	-0.7 to -0.65	1,732	3.4%	0.9 to 0.95	3,109	6.1%
	-0.65 to -0.6	1,984	4.0%	0.95 to 0.99	3,432	6.8%
	-0.6 to -0.55	2,226	4.4%	>0.99	5,387	10.6%
	-0.55 to -0.5	2,244	4.4%			
	>-0.5	6,363	12.5%			
Total	-	50,750	100%	-	50,750	100%

Table S4: **JWRs remaining wrong after NanoSplicer correction**

take into account the uncertainty of the summary values, (note that NanoSplicer partitions an observed squiggle into multiple segments, combines noisy measurements of the squiggle into a more stable summary value at each segment, and use the summary values as data in the NanoSplicer model; see section 2.4.1 in the main text for details). One potential way to incorporate the uncertainty is to exploit the likelihood approximation as described in Shim *et al.* (2021), yielding likelihoods expressed by the estimates of model parameters and their standard errors. We leave it for future work.

2.12 More examples of splice junction correction with NanoSplicer

To demonstrate how squiggles can provide extra information to identify splice junctions, Supplementary Fig. S15 A1 & A2 and Fig. S15 B1 & B2 show examples of splice junction correction with NanoSplicer from the synthetic and biological datasets, respectively.

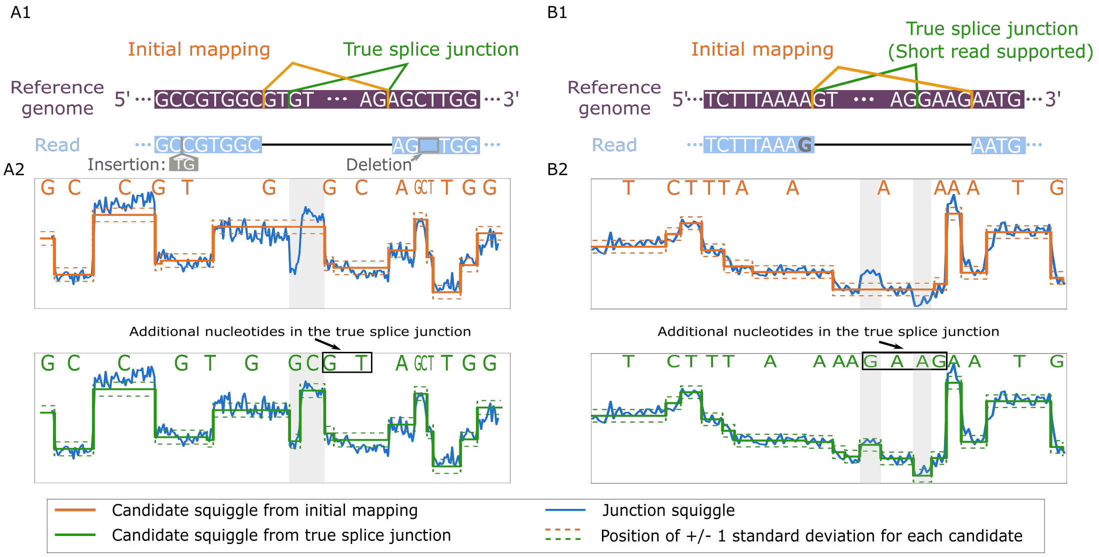

Fig. S15: **More examples of NanoSplicer correcting wrongly mapped JWRs** A1 & A2: Synthetic RNA data, B1 & B2: Biological data. A1 & B1: JWR mapping. Reference genome sequence shown in purple. Green line shows the location of the known ground truth splice junction. The mapped nanopore read (blue) shows the basecalled nucleotides of the JWR and how they were aligned to the reference genome. Mismatched nucleotide is highlighted by dark grey. Orange line shows the splice junction identified by the initial mapping of the JWR. Insertion - basecalled nucleotides in read that were not part of genome alignment. Deletion - nucleotides in the reference genome that are not part of the read. A2 & B2: Alignments between the junction squiggle for the JWR (blue) and the corresponding candidate squiggles from A1 & B1 (orange and green). Each junction squiggle current measurement is vertically aligned with its assigned mean-standard deviation in the candidate squiggle. The junction motifs for each candidate are shown at the top of each panel. Each nucleotide in the motifs is aligned with its corresponding squiggle position. Panels focus on the junction squiggle areas that distinguish between the candidates (grey background; the absolute difference in log likelihood between the two candidate models is bigger than 1.35; see Supplementary section 2.13 for details).

2.12.1 Additional example in synthetic RNA data

In the reference genome in Supplementary Fig. S15A1, there are two "GT" 5' splice motifs right next to each other, leading to two candidate splice junctions. Based on the ground truth of the synthetic RNA, two exonic bases "GT" preceding the 5' splice site were deleted from the nanopore read, resulting in the JWR initially mapped to the wrong 5' splice site with a 2-base shift. We compared the junction squiggle of this JWR to the candidate squiggles obtained from the true splice junction and the splice junction matching the initial mapping. Supplementary Fig. S15A2 shows the alignments between the junction squiggle and the two candidate squiggles respectively. The squiggle from the true candidate splice junction is visually a closer match to the junction squiggle. NanoSplicer quantified this squiggle similarity, giving an assignment probability of 0.99 to the true candidate for this JWR.

2.12.2 Additional example in biological RNA data

In the reference genome in Supplementary Fig. S15B1, there are two "AG" 3' splice motifs 4 bases apart at the 3' splice site, leading to two candidate splice junctions. One of the candidate splice junctions is supported by the short read data and is assumed to be the true one. However, the nanopore read in supplementary Fig. S15B1 was mapped to the other candidate splice junction due to basecalling errors near the splice junction (the *"A"* and *"GAAG"* preceding and after the true splice junction were basecalled as only *"G"* in the nanopore read). We compared the junction squiggle of this JWR to the squiggles obtained from both candidate splice junctions. Supplementary Fig. S15B2 shows the alignments between the junction squiggle and the two candidate squiggles respectively. The shape of the squiggle for the true candidate is clearly a better match for the junction squiggle. NanoSplicer quantified this squiggle similarity, leading to an assignment probability of 0.999 to the true candidate for this JWR.

2.13 Identification of junction squiggle areas that distinguish between two candidates

In Fig. 3 in the main text and Fig. S15 for examples of NanoSplicer correcting wrongly mapped JWRs, we highlighted junction squiggle areas that distinguish between two candidates using grey background. We identified those areas as follows. Let y_n represent a summary value at the n-th segment of the junction squiggle. Suppose y_n is aligned to (μ^t, σ^t) and (μ^o, σ^o) in the candidate squiggles $\boldsymbol{c}_s^t$ and $\boldsymbol{c}_s^o$, respectively. Summary values from the same squiggle (i.e., the same read) share the same standard deviation, so $\sigma^t = \sigma^o = \sigma$. Assuming that $\boldsymbol{c}_s^t$ is the candidate squiggle identified by NanoSplicer, we denote μ^t by $\mu^o + \tilde{\mu}$. Then, $y_n \sim \mathcal{N}^*(\mu^o, \sigma)$ for the candidate squiggle $\boldsymbol{c}_s^o$ and $y_n \sim \mathcal{N}^*(\mu^o + \tilde{\mu}, \sigma)$ for the candidate squiggle $\boldsymbol{c}_s^t$, where $\mathcal{N}^*$ denotes the modified normal distributions. Then, the n-th segment is junction squiggle areas that distinguish between the two candidates if

$$-2\left[\log f(y_n|\mu^o, \sigma) - \log f(y_n|\mu^o + \tilde{\mu}, \sigma)\right] > \chi^2_{1,\alpha}, \tag{11}$$

where f is the probability density function of the modified normal distributions (see Supplementary section 1.5 for details) and $\chi^2_{1,\alpha}$ represents the $1 - \alpha$ quantile for the χ^2 distribution with 1 degree of freedom. We used $\alpha = 0.1$ in all figures of this paper.

2.14 Run time

The NanoSplicer analysis pipeline includes following steps: 1. Identify the locations of JWRs from mapped reads; 2. Calculate junction alignment quality (JAQ) for each JWR; 3. Perform splice junction identification for each JWR. In steps 2 and 3, the entire analysis is parallelizable because the analysis for each JWR is independent. Multiprocessing is available in NanoSplicer (`https://github.com/shimlab/NanoSplicer`). Table S5 shows the run time for each step in our analysis of synthetic and biological datasets.

Table S6 shows the run time of FLAIR and StringTie2 for biological data analysis. Their run time is shorter compared to NanoSplicer, although this is not unexpected given the higher computational burden of NanoSplicer analysis. A higher computational burden is a common trade-off of methods based on probabilistic models such as NanoSplicer. Note that splice junction identifications in FLAIR and StringTie2 are not based on probabilistic models. However, due to the flexible nature of NanoSplicer, it can be run on JWRs of interest and not just transcriptome-wide. For example, it can be focused only on JWRs below a user-specified JAQ threshold, or on genes and genomics regions of interest. This will greatly shorten run time, while still providing the information required by the user.

	**Identify the location of JWRs**	**Calculate junction alignment quality for all JWRs**	**Identify splice junctions for all JWRs**
Synthetic RNA dataset	∼2 mins	∼7.8 CPU hours (∼16 mins with 32 CPUs)	∼7151 CPU hours (∼124 hours with 72 CPUs)
Biological RNA dataset	∼1.5 mins	∼2.5 CPU hours (∼6 mins with 32 CPUs)	∼2007 CPU hours (∼35 hours with 72 CPUs)

Table S5: **NanoSplicer run time** The numbers in the table represent NanoSplicer run time for analysis of 3,526,941 and 2,220,118 JWRs from 1,525,817 and 758,009 mapped reads in the synthetic and biological RNA datasets, respectively. When multiprocessing option is available, the numbers in brackets represent NanoSplicer run time with the number of CPUs used in our analysis, and the numbers outside the brackets are the total CPUs hours.

	**FLAIR (flair correct module)**	**StringTie2**
Biological RNA dataset	∼30 seconds	∼20 seconds

Table S6: **FLAIR and StringTie2 run time (CPU time)** We ran FLAIR (splice site correction step) and StringTie2 on 758,330 mapped reads (BAM file) in the biological data. Note that the run time for FLAIR is for "flair correct" module, which is the module for the splice site correction in the FLAIR package.

2.15 Impact of including common non-canonical junctions as candidates

NanoSplicer provides the option of including "GC-AG" and "AT-AC" junctions as candidates, as they are the most prevalent non GT-AG junctions in mammals (∼0.7% and ∼0.05% of splice junctions (Burset, 2000)). Using this option, we have added all GC-AG/AT-AC junctions within 10 bases of each JWR to the candidates initially selected by using the default option ("default

candidates"; see section 4 in the main text for details) and re-applied NanoSplicer to the biological data. In this section, we summarise the impact of including GC-AG/AT-AC junctions as candidates by comparing the performance of NanoSplicer with the default candidates and the default candidates + GC-AG/AT-AC junctions; see Table S7.

Adding GC-AG/AT-AC junctions as candidates significantly improved the identification of JWRs with non-canonical ground truth (accuracy increased from 49.7% to 83.8%), because 98.1% of those JWRs in the dataset used GC-AG or AT-AC junctions.

However, the overall impact on NanoSplicer accuracy from including common non-canonical junctions is slight (from 97.1% to 97.2%). Therefore, we don't include GC-AG/AT-AC junctions as candidates by default because it roughly doubles the runtime of NanoSplicer (runtime for each JWR increased from 3 to 6 sec) without much benefit on overall accuracy. Even so, if accurate identification of GC-AG and AT-AC junctions is important, users can use the option to do so, which we would recommend when users need higher accuracy for non GT-AG junctions or are analyzing genes that utilise non GT-AG junctions.

	Overall accuracy (calculation from 100K randomly selected JWRs)	Accuracy of JWRs whose ground truths are non-canonical splice junctions (8,861 JWRs in total)	Accuracy for JWRs whose ground truths are GC-AG/AT-AC junctions (8,689 JWRs in total)	Average number of candidates (runtime) per each JWR
Default candidates	97.1% (91,922 JWRs)	49.7% (7,373 JWRs)	49.8% (7,233 JWRs)	3.3 (runtime: 3.3s)
Default candidates + GC-AG/AT-AC junctions	97.2% (92,053 JWRs)	83.8% (6,323 JWRs)	84.8% (6,181 JWRs)	5.5 (runtime: 5.9s)

Table S7: **Performance of NanoSplicer with the default candidates and the default candidates + GC-AG/AT-AC junctions** The numbers in the brackets represent the numbers of JWRs after SIQ and strongest probability filtering in NanoSplicer.

2.16 Improved performance of NanoSplicer in rarer junction identification compared to methods that correct splice junctions using information from other mapped reads

Methods such as StringTie2 (Kovaka *et al.*, 2019) correct splice junctions using information from other mapped reads (e.g., nearby splice junctions supported by high read counts), potentially leading less abundant junctions to go undetected. Compared to those methods, NanoS-

plicer enables its identification to be independent of other reads, possibly having the advantage in rarer junction identification. To demonstrate this advantage, we compared NanoSplicer with StringTie2 at all sites where we could ascertain if rarer splice junctions were being replaced. Specifically, using the biological short read dataset (see supplementary sections 2.1 and 2.4 for details of the dataset), we first considered splice junctions with at least 3 mapped short reads to be "true". We then focused on 492 sites where there are multiple nearby true splice junctions. That is, each site has multiple true junctions whose 5' or 3' splice sites are $\leq$10nt away from that of another junctions in the site. We will refer to these multiple junctions as a "junction set". To assess the ability of each method to identify rarer junctions, we compared the number of a "fully detected junction set", defined by a junction set where all true junctions are detected by each method using the biological nanopore sequencing dataset (see supplementary sections 2.1, 2.2, and 2.3 for details of the dataset). Among the 492 junction sets, StringTie2 fully detected 10 junction sets (2%) while NanoSplicer was successful for 273 (55.5%). This result illustrates the benefits of NanoSplicer, whose performance is independent of other reads, in identifying rare junctions. We observed similar results when true splice junctions were defined by using different minimum numbers of short reads (supplementary Table S8).

Minimum number of short reads to define true splice junctions	Junction sets identified by short reads	Junction sets fully detected by NanoSplicer	Junction sets fully detected by StringTie2 (default setting; high sensitivity setting)
3	492	273	10; 15
10	201	149	10; 13
20	116	96	10; 12

Table S8: **The number of junction sets fully detected by NanoSplicer and StringTie2.** This table shows, for each minimum number of short reads to define true splice junctions, the number of junction sets constructed by the true splice junctions and the numbers of fully detected junction sets by NanoSplicer and StringTie2. StringTie2 has been applied with two different settings: 1. default long read setting (command "stringtie -L") whose results have been presented in the main text; 2. high sensitivity setting (command "stringtie -L -f 0 -m 30 -t -E 10") which uses relaxed thresholds for the final output to increase the sensitivity of splice junction detection.

Note that NanoSplicer identifies splice junctions only for JWRs whose squiggles are informative, limiting the potential usage of its output in splice junction "quantification" (see main text section 5 for more discussion). Therefore, we assessed the ability of each method to identify rarer junctions using the number of fully detected junction sets, rather than performing splice junction quantification.

2.16.1 Example of StringTie2 replacing rarer splice junctions with those from more highly expressed isoforms

This section presents one example of junction sets fully detected by NanoSplicer but not by StingTie2, i.e., example of StringTie2 replacing rarer splice junctions with those from more highly expressed isoforms (supplementary Fig. S16). In this example, there are two different splice junctions supported by 63 and 146 short reads, respectively. Short reads are expected to provide accurate information on the locations of splice junctions, supporting that these two splice junctions are real. The 3' splice site in the minor splice junction (one supported by 63 short reads) is different in 3 nucleotides towards the upstream of the major one (supported by 146 short reads); see supplementary Fig. S16A. The initial mapping of long reads support both splice junctions (21 and 11 long reads mapped to the major and minor splice junctions, respectively). However, all isoforms assembled by StringTie2 using these mapped long reads support only the major splice junction, leading to the minor splice junction being undetected (supplementary Fig. S16C).

Supplementary Fig. S16D shows splice junctions identified by NanoSplicer using the same dataset (see main text section 4 for the details). NanoSplicer identified both splice junctions (the major and minor splice junctions are identified at 20 and 7 JWRs, respectively; NanoSplicer did not provide identification results for 5 JWRs due to their poor squiggle information quality; see main text section 4 for the details). This supports that NanoSplicer has a good potential for identifying rare splice junctions as its performance is not affected by other reads.

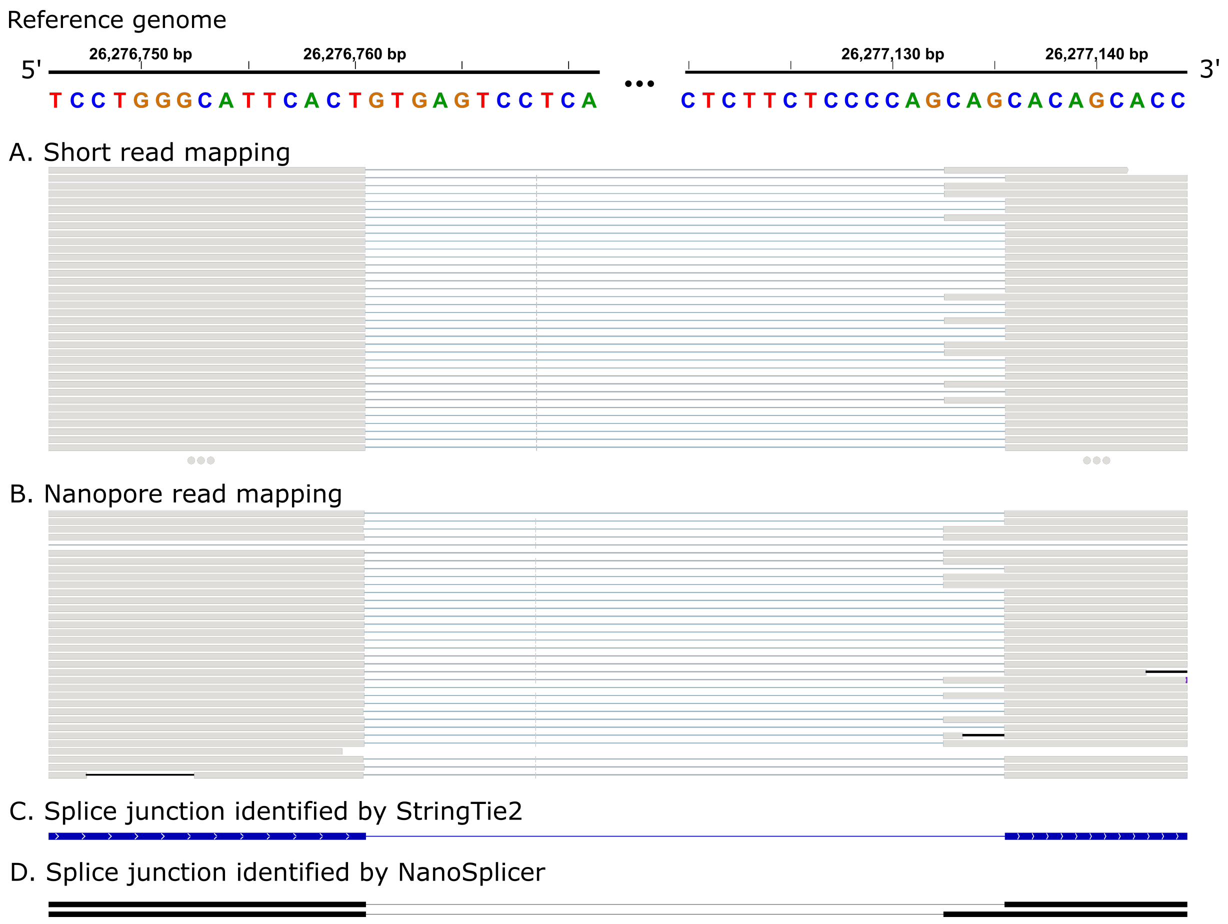

Fig. S16: **Example of StringTie2 replacing rarer splice junctions with those from more highly expressed isoforms.** The genome region in the figure is 26,276,746-26,277,144 bp on chromosome 1 in the human genome (GRCh38). See supplementary section 2.1 for the detailed description of the sequencing data used in this figure. Note that this figure has been created by simplifying a screenshot of the Integrative Genomics Viewer (IGV) (Robinson *et al.*, 2011). A. Short read mapping result at the corresponding region. Note that only a proportion of the mapped short reads is displayed. B. Long read mapping result at the corresponding region. C. Splice junction for all isoforms assembled by StringTie2 using the long reads. D. Splice junctions identified by NanoSplicer using the long reads.

2.17 Improved performance of NanoSplicer when there are multiple nearby annotated junctions, or the true junctions utilise unannotated splice sites

Methods such as FLAIR (Tang *et al.*, 2020) require a set of splice junctions, either from annotations or from matched short reads, to be provided to guide their corrections. While NanoSplicer

does not require those input splice junctions, it provides the option to utilise them, for example, in organisms where comprehensive annotations are available. We compared the output of FLAIR with NanoSplicer, including human GENCODE v39 annotations as input, to understand the relative performance of each on different categories of JWRs. Note that we used only annotations as input because the matched short reads have been used to define the ground truths for assessment of the performance (see section 4 in the main text).

We first review differences in functionalities between NanoSplicer and FLAIR in supplementary section 2.17.1, and then present results from the comparison in supplementary section 2.17.2. Note that the "FLAIR" in this section indicates only the "FLAIR correct" module (i.e., splice site correction module in FLAIR) for simplicity. There are other modules available in FLAIR for different purposes.

2.17.1 Difference in functionalities between NanoSplicer and FLAIR

NanoSplicer and FLAIR correct splice junctions from mapped long reads. FLAIR requires a set of splice junctions, either from annotations or from matched short reads, as input, and corrects all JWRs to splice junctions/sites in this set. However, this set is an optional input to NansoSplicer which can correct JWRs solely from nanopore data without using annotations or matched short reads. Moreover, when the set of junctions is provided, two methods use it differently. FLAIR corrects JWRs only to the provided splice junctions/sites, failing to find novel splice junctions. However, NanoSplicer can include the provided splice junctions as candidates "in addition to" default candidates, still enabling it to identify novel junctions if they are included as the default candidates and supported by squiggle matching. Also, when multiple nearby junctions from the input set are available for correction, FLAIR chooses one closest to a mapped junction by default, while NanoSplicer uses information in squiggles.

2.17.2 Comparison of NanoSplicer and FLAIR

We applied NanSplicer and FLAIR to the biological RNA dataset (see section 4 in the main text and Supplementary section 2.1), including the human GENCODE v39 annotations as input. For the purpose of assessment, we defined a ground truth for each JWR using the matched short read data (see section 4 in the main text and Supplementary section 2.1). For each JWR, we searched for a junction supported by the short reads within 10nt of the mapped splice sites and treated it as a ground truth for that JWR. To avoid ambiguity in determining the ground truth, we restricted our assessment to 1,880,011 JWRs that had at most one nearby splice junction supported by the short reads. We used the default setting for FLAIR, and considered the following three modes for NanoSplicer using its flexible options for the candidate selection:

1. "no annotation": default candidates (i.e., mapped splice junctions and all GT-AG junctions within 10nt)
2. "with annotation": default candidates + *annotated junctions* from the human GENCODE v39 annotations
3. "annotation only": *annotated junctions* from the human GENCODE v39 annotations.

Supplementary section 1.9 provides the definition of *annotated junctions* in NanoSplicer and explains prior mixing proportions for the annotated junctions.

Note that NanoSplicer with "annotation only" mode only uses the annotated junctions as candidates and hence it corrects JWRs only to them, failing to identify novel splice junctions (like FLAIR). In this mode, JWRs with no annotated junctions within 10nt (no candidate) are filtered out. When JWRs have only one annotated junction within 10nt (only one candidate), NanoSplicer uses it for correction without considering squiggle information, i.e., returning the only annotated one as an identified junction without considering SIQ filtering. For JWRs with

multiple nearby annotated junctions (multiple candidates), NanoSplicer uses squiggle information for correction, and it performs the SIQ and assignment probability filtering because it can choose one among candidates only when squiggles are informative.

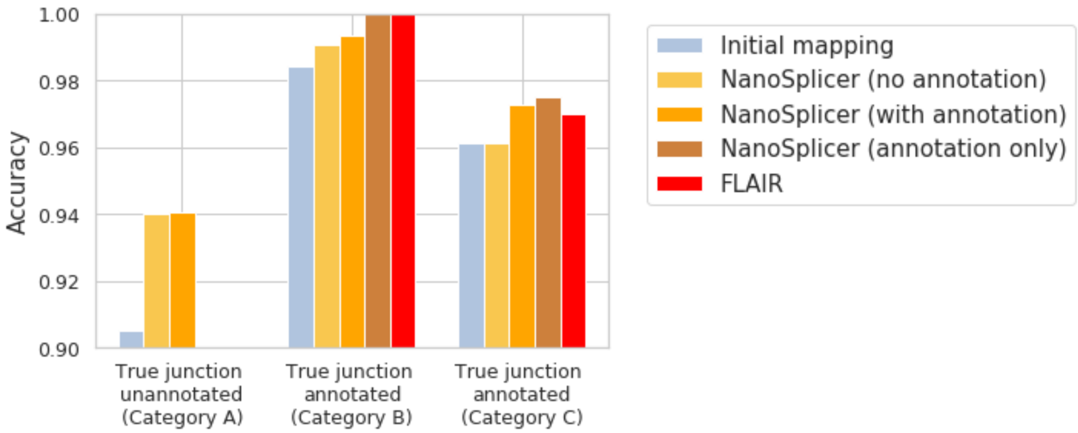

Fig. S17: **Accuracy of NanoSplicer with three different modes, FLAIR, and initial mapping for three categories of JWRs.** JWRs in category A have unannotated junctions (novel junctions) as ground truths (6,385 JWRs); JWRs in category B have annotated junctions as ground truths and have only one annotated junction within 10nt (1,780,475 JWRs); JWRs in category C have annotated junctions as ground truths and have multiple annotated junctions within 10nt (40,982 JWRs). Note that except for the "annotation only" mode, NanoSplicer includes the default candidate (GT-AG junction + mapped junction).

Then, we assess the relative performance of each approach on the following four categories of JWRs (Figure S17 and Table S9).

A. JWR whose ground truth is unannotated, i.e., being novel (6,385 JWRs): For JWRs in this category, FLAIR and NanoSplicer with "annotation only" mode output no identified junctions if there is no annotated junction within 10nt. Otherwise, they correct the JWRs to annotated junctions. Thus, they fail to identify unannotated ground truths, resulting in an accuracy of 0%. However, NanoSplicer with "no annotation" or "with annotation" mode still includes default candidates, enabling it to identify ground truths if they are included as default candidates and supported by squiggle matching. NanoS-

plicer in these two modes identified ground truths for ∼94% of the JWRs in this category, leading to the improved accuracy compared to the initial mapping (Figure S17).

B. **JWR whose ground truth is annotated and is the only annotated junction within 10nt (1,780,475 JWRs):** For JWRs in this category, FLAIR and NanoSplicer with "annotation only" mode correct all JWRs to ground truths because they are the only annotated junctions within 10nt, resulting in an accuracy of 100%. However, NanoSplicer with "no annotation" or "with annotation" mode considers other (unannotated) junctions as default candidates if they exist within 10nt. Moreover, NanoSplicer with "no annotation" can only consider the ground truths as candidates if they are included as default candidates (i.e., mapped splice junctions and all GT-AG junctions). Nevertheless, NanoSplicer in these two modes identified ground truths with a high accuracy of ∼99%, resulting in the improved accuracy compared to the initial mapping (Figure S17).

C. **JWR whose ground truth is annotated and that has multiple annotated junctions within 10nt – the ground truth is not the only annotated junction within 10nt (40,982 JWRs):** JWRs in this category have multiple nearby annotated junctions for the correction of FLAIR and NanoSplicer with "annotation only" mode, and one of them is the ground truth. FLAIR chooses one closest to the mapped junctions by default, while NanoSplicer with "annotation only" mode uses information in squiggles for correction. NanoSplicer with "with annotation" mode also has multiple candidates (including the annotated ones) and identifies one using squiggle information. Thus, NanoSplicer with "annotation only" and "with annotation" modes provides increased accuracy compared to FLAIR (Figure S17). Fig. S18 shows an example of JWRs in this category where the ground truth is identified by NanoSplicer with "annotation only" or "with annotation" mode, but missed by FLAIR. The JWR in this example has 2 annotated splice junc-

tions within 10nt of the mapped junction and FLAIR identified the wrong one which is closer to the initially mapped junction. However, the expected squiggle from the ground truth is visually a closer match to the junction squiggle and NanoSplicer quantified this squiggle similarity, successfully identifying the ground truth with an assignment probability of 0.988. NanoSplicer with "no annotation" can only consider the ground truths as candidates if they are included as default candidates (i.e., mapped splice junctions and all GT-AG junctions). NanoSplicer in this mode shows a lower accuracy compared to NanoSplicer with other modes and FLAIR, and shows similar accuracy compared to the initial mapping (Figure S17).

D. **Completely missed JWR (52,169 JWRs):** Completely missed JWRs are those without junctions supported by short reads within 10nt, having no ground truths. Any identification by NanoSplicer and FLAIR for the completely missed JWRs will be incorrect, but both methods can filter out some of them, affecting overall accuracy. NanoSplicer with "annotation only" mode and FLAIR identify splice junctions only for JWRs that have annotated junctions within 10nt, and most ground truths are annotated junctions in this dataset (see the number of JWRs in each category). Thus, they filtered out more completely missed JWRs compared to NanoSplicer with other modes (Table S9). Note that FLAIR filtered out more JWRs (so more completely missed JWRs) compared to NanoSplicer with "annotation only" mode because FLAIR removes entire reads from its identification of splice junctions if they contain at least one JWR without annotated junctions within 10nt.

In summary, compared to FLAIR, NanoSplicer provides improved performance when there are multiple annotated junctions within 10nt of the JWR, or the ground truth utilised unannotated splice junctions (Figure S17). Particularly, Nanosplicer with "annotation only" mode

	Number of JWRs	Number (proportion) of completely missed JWRs
**Initial mapping**	1,880,011	52,169 (2.8%)
**NanoSplicer (no annotation)**	1,724,883	33,604 (2.0%)
**NanoSplicer (with annotation)**	1,732,291	33,622 (1.9%)
**NanoSplicer (annotation only)**	1,830,980	12,273 (0.7%)
**FLAIR**	1,717,772	9,678 (0.6%)

Table S9: **Numbers of (all) JWRs and completely missed JWRs where each method provides identified splice junctions after its own filtering**

outperforms FLAIR when there are multiple nearby annotated junctions while giving equivalent results otherwise. Overall accuracy of each method depends on the relative proportion of JWRs in each category. Also, the relative proportion is heavily dependent on the quality of the annotation of an organism or biological system. For example, for a non-model organism or in disease-causing altered mRNA processing where suitable annotations may not be available, the proportion of the category A is large, leading to increased overall accuracy of NanSplicer with "no annotation" or "with annotation" mode.

Comparison of NanoSplicer and FLAIR for JWRs with multiple ground truths within 10nt: The 1,880,011 JWRs belonging to the four categories above have at most one ground truth within 10nt. To assess the performance of each method for JWRs with multiple ground truths within 10nt, we performed an analysis similar to one in Supplementary section 2.16. Specifically, we assessed the performance of each method by using the number of fully detected junction sets (out of the 492 junction sets; see Supplementary section 2.16 for the definitions of junction set and fully detected junction set); see Table S10 for results of the analysis. NanoSplicer with "annotation only" mode and FLAIR cannot fully detect any of 287 junction sets that include at least one unannotated junction, resulting in 141 and 139 fully detected junction sets, respectively. NanoSplicer with "no annotation" and "with annotation" modes fully detected more junction sets (273 and 277, respectively) due to their ability to identify unannotated junctions.

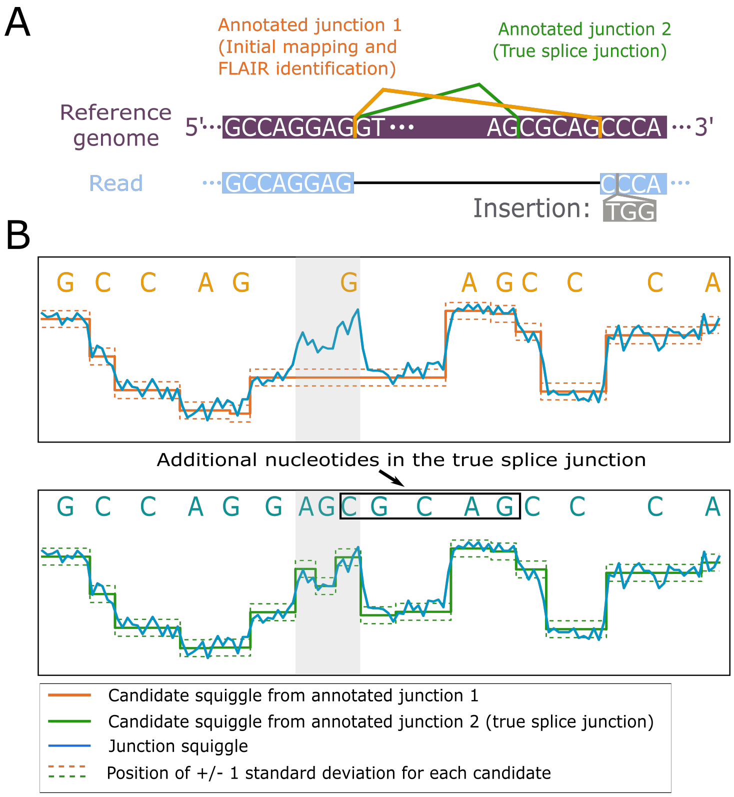

Fig. S18: **Example of JWRs in the category C where the ground truth is identified by NanoSplicer with "annotation only" or "with annotation" mode, but missed by FLAIR** A: JWR mapping. The reference genome sequence is shown in purple. The mapped nanopore read (blue) shows the basecalled nucleotides of the JWR and how they were aligned to the reference genome. The green and orange lines show the locations of two annotated splice junctions (1 and 2). Annotated junction 1 (orange line) is the splice junction identified by the initial mapping and FLAIR and the annotated junction 2 (green line) is the ground truth splice junction (i.e., the only nearby splice junction supported by short reads). Insertion - basecalled nucleotides in the read that are not part of genome alignment. B: Alignments between the junction squiggle for the JWR (blue) and the corresponding candidate squiggles from A (orange and green). Each junction squiggle current measurement is vertically aligned with its assigned mean-standard deviation in the candidate squiggle. The junction motifs for each candidate are shown at the top of each panel. Each nucleotide in the motifs is aligned with its corresponding squiggle position. Panels focus on the junction squiggle areas that distinguish between the candidates (grey background; the absolute difference in log-likelihood between the two candidate models is bigger than 1.35; see Supplementary section 2.13 for details).

	Number of junction sets (from short reads)	NanoSplicer "no annotation"	NanoSplicer "with annotation"	NanoSplicer "annotation only"	FLAIR
**Junction set consisting of annotated junctions**	205	137	141	141	139
**Junction set with at least one unannotated junction**	287	136	137	0	0
**Total**	492	273	277	141	139

Table S10: **The number of junction sets fully detected by NanoSplicer and FLAIR.** This table shows the number of junction sets constructed by the true splice junctions (junction sets consisting of annotated junctions and junction sets containing at least one unannotated junction) and the numbers of fully detected junction sets by NanoSplicer with three different modes and FLAIR. Note that "fully detected junction set" is defined by a junction set where all true junctions are detected by each method.

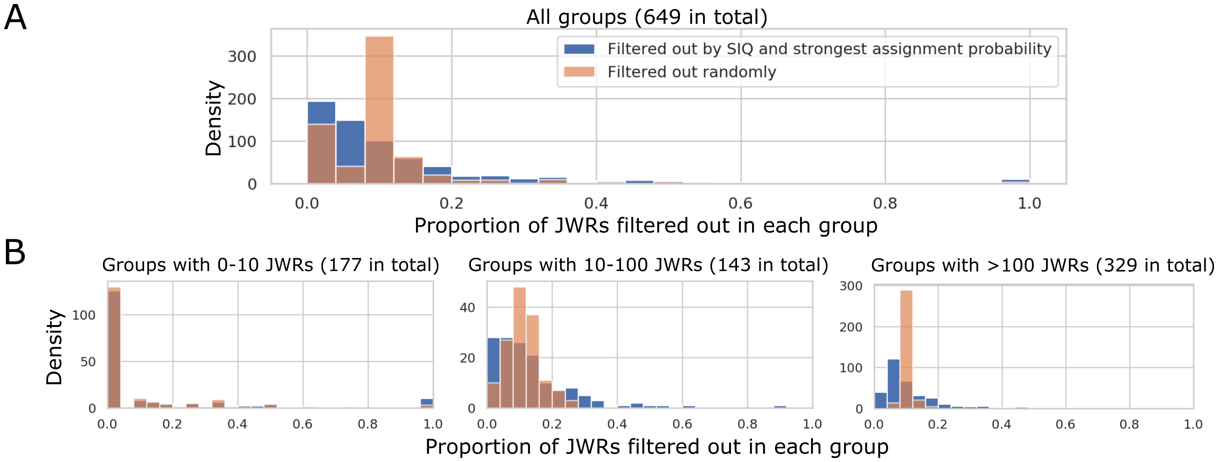

Fig. S19: **JWRs filtered out by SIQ and strongest assignment probability are unlikely to be a random subset of all JWRs.** In the synthetic data analysis (section 3 in the main text), we identified 649 unique sequins splice junctions as ground truths for 3,333,887 JWRs (not included the completely missed JWRs). We grouped the JWRs based on their ground truths, leading to 649 groups of JWRs. JWRs in each group share the same ground truth. Out of the 3,333,887 JWRs, 349,703 JWRs have been filtered out by using SIQ and strongest assignment probability in our analysis of section 3. For each group, we computed the proportion of the filtered JWRs. Panel A shows the empirical distribution of that proportions constructed using all 649 groups (blue) and panel B shows those distributions for different sets of groups with different ranges in their size (i.e., the number of JWRs). To compare those distributions with that when a random subset of JWRs is filtered out, we randomly selected 349,703 JWRs from the 3,333,887 JWRs, filtered out them, computed the proportions of the filtered JWRs, and plotted similar empirical distributions (in orange in panels A and B). As expected, these distributions for the random filtering (orange) have modes around 0.1, showing similar proportions across groups (except for groups with 0-10 JWRs which have noisy proportion estimates). Compared to that (i.e., expectation by chance), the proportions after the SIQ and strongest assignment probability filtering showed higher variations (i.e., wider distributions), supporting that JWRs filtered out by these filtering criteria are unlikely to be a random subset of all JWRs. Note that the group with 0-10 JWRs have noisy proportion estimates, showing distributions different from the expectation.

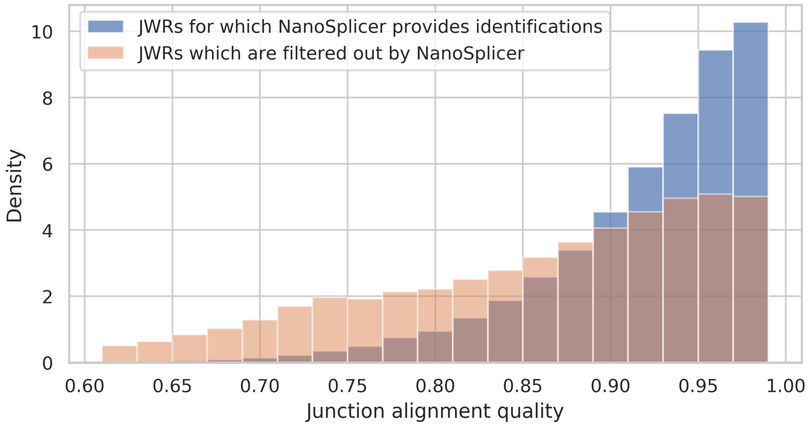

Fig. S20: **Distributions of JAQ for JWRs filtered out / remaining.** In our synthetic data analysis (section 3 in the main text), 460,060 JWRs are filtered out by NanoSplicer. Figure shows two distributions of junction alignment quality (JAQ) for the 460,060 JWRs filtered out and the remaining 3,066,881 JWRs.

References

Ast, G. (2004). How did alternative splicing evolve? *Nature Reviews Genetics*, **5**(10), 773–782.

Bolger, A. M., Lohse, M., and Usadel, B. (2014). Trimmomatic: a flexible trimmer for illumina sequence data. *Bioinformatics*, **30**(15), 2114–2120.

Burset, M. (2000). Analysis of canonical and non-canonical splice sites in mammalian genomes.

Dobin, A., Davis, C. A., Schlesinger, F., Drenkow, J., Zaleski, C., Jha, S., Batut, P., Chaisson, M., and Gingeras, T. R. (2013). Star: ultrafast universal rna-seq aligner. *Bioinformatics*, **29**(1), 15–21.

Dong, X., Tian, L., Gouil, Q., Kariyawasam, H., Su, S., De Paoli-Iseppi, R., Prawer, Y. D. J., Clark, M. B., Breslin, K., Iminitoff, M., *et al.* (2021). The long and the short of it: unlocking nanopore long-read rna sequencing data with short-read differential expression analysis tools. *NAR genomics and bioinformatics*, **3**(2), lqab028.

Frankish, A., Diekhans, M., Jungreis, I., Lagarde, J., Loveland, J. E., Mudge, J. M., Sisu, C., Wright, J. C., Armstrong, J., Barnes, I., *et al.* (2021). Gencode 2021. *Nucleic acids research*, **49**(D1), D916–D923.

Holik, A. Z., Law, C. W., Liu, R., Wang, Z., Wang, W., Ahn, J., Asselin-Labat, M.-L., Smyth, G. K., and Ritchie, M. E. (2017). Rna-seq mixology: designing realistic control experiments to compare protocols and analysis methods. *Nucleic acids research*, **45**(5), e30–e30.

Irimia, M. and Roy, S. W. (2008). Evolutionary convergence on highly-conserved 3 intron structures in intron-poor eukaryotes and insights into the ancestral eukaryotic genome. *PLoS Genet*, **4**(8), e1000148.

Keogh, E. and Ratanamahatana, C. A. (2005). Exact indexing of dynamic time warping. *Knowledge and information systems*, **7**(3), 358–386.

Kovaka, S., Zimin, A. V., Pertea, G. M., Razaghi, R., Salzberg, S. L., and Pertea, M. (2019). Transcriptome assembly from long-read rna-seq alignments with stringtie2. *Genome biology*, **20**(1), 1–13.

Li, H. (2017). Which human reference genome to use?, `https://lh3.github.io/2017/11/13/which-human-reference-genome-to-use`.

Li, H. (2018). Minimap2: pairwise alignment for nucleotide sequences. *Bioinformatics*, **34**(18), 3094–3100.

Rang, F. J., Kloosterman, W. P., and de Ridder, J. (2018). From squiggle to basepair: computational approaches for improving nanopore sequencing read accuracy. *Genome biology*, **19**(1), 1–11.

Robinson, J. T., Thorvaldsdóttir, H., Winckler, W., Guttman, M., Lander, E. S., Getz, G., and Mesirov, J. P. (2011). Integrative genomics viewer. *Nature biotechnology*, **29**(1), 24–26.

Shim, H., Xing, Z., Pantaleo, E., Luca, F., Pique-Regi, R., and Stephens, M. (2021). Multi-scale poisson process approaches for differential expression analysis of high-throughput sequencing data. *arXiv preprint arXiv:2106.13634*.

Tang, A. D., Soulette, C. M., van Baren, M. J., Hart, K., Hrabeta-Robinson, E., Wu, C. J., and Brooks, A. N. (2020). Full-length transcript characterization of sf3b1 mutation in chronic lymphocytic leukemia reveals downregulation of retained introns. *Nature communications*, **11**(1), 1–12.
